# Probing RNA Conformations Using a Polymer–Electrolyte Solid-State Nanopore

**DOI:** 10.1021/acsnano.2c08312

**Published:** 2022-10-24

**Authors:** Chalmers Chau, Fabio Marcuccio, Dimitrios Soulias, Martin Andrew Edwards, Andrew Tuplin, Sheena E. Radford, Eric Hewitt, Paolo Actis

**Affiliations:** † School of Molecular and Cellular Biology and Astbury Centre for Structural Molecular Biology, University of Leeds, Leeds LS2 9JT, U.K.; ‡ School of Electronic and Electrical Engineering and Pollard Institute, University of Leeds, Leeds LS2 9JT, U.K.; § Bragg Centre for Materials Research, University of Leeds, Leeds LS2 9JT, U.K.; ∥ Department of Chemistry & Biochemistry, University of Arkansas, Fayetteville, Arkansas 72701, United States

**Keywords:** nanopore, RNA, DNA, single molecule, nanopipette, PEG, polymer−electrolyte

## Abstract

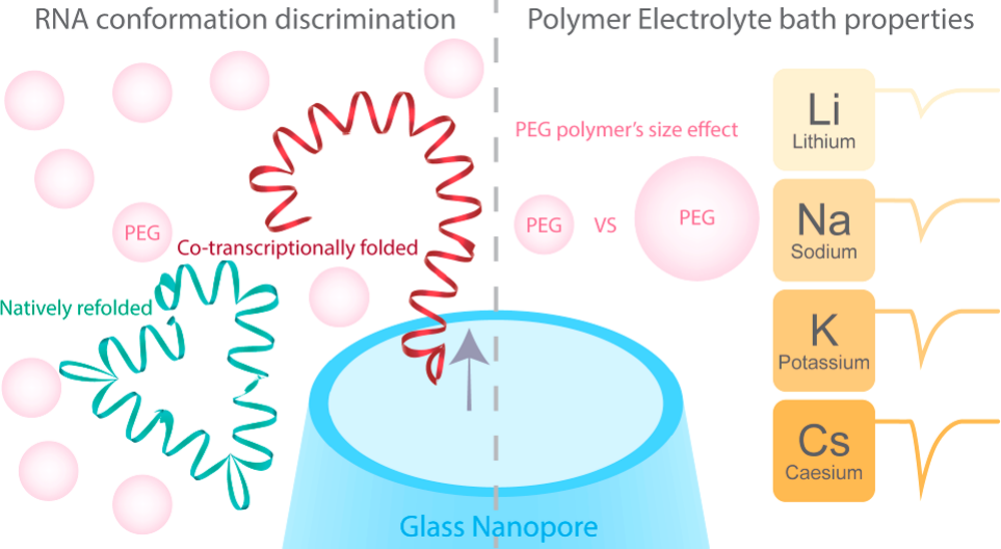

Nanopore systems have emerged as a leading platform for the analysis of biomolecular complexes with single-molecule resolution. The conformation of biomolecules, such as RNA, is highly dependent on the electrolyte composition, but solid-state nanopore systems often require high salt concentration to operate, precluding analysis of macromolecular conformations under physiologically relevant conditions. Here, we report the implementation of a polymer–electrolyte solid-state nanopore system based on alkali metal halide salts dissolved in 50% w/v poly(ethylene) glycol (PEG) to augment the performance of our system. We show that polymer–electrolyte bath governs the translocation dynamics of the analyte which correlates with the physical properties of the salt used in the bath. This allowed us to identify CsBr as the optimal salt to complement PEG to generate the largest signal enhancement. Harnessing the effects of the polymer–electrolyte, we probed the conformations of the Chikungunya virus (CHIKV) RNA genome fragments under physiologically relevant conditions. Our system was able to fingerprint CHIKV RNA fragments ranging from ∼300 to ∼2000 nt length and subsequently distinguish conformations between the co-transcriptionally folded and the natively refolded ∼2000 nt CHIKV RNA. We envision that the polymer–electrolyte solid-state nanopore system will further enable structural and conformational analyses of individual biomolecules under physiologically relevant conditions.

## Introduction

Nanopore technology enables the analysis of biological macromolecules with single-molecule resolution.^1,2^ In nanopore experiments, individual molecules are driven through a nanopore under the influence of an electric field, causing a temporary modulation in the conductance within the pore produced by a combination of the geometrical exclusion of solution, ion concentration polarization and additional charges brought by the analyte itself.^3,4^ The magnitude and duration of this momentary change in the ionic current reflects the translocation dynamics of the molecule, which are dependent on the properties of the molecule (*e.g.*, size, shape, charge).^5−20^ There are two classes of nanopores: biological nanopores and solid-state nanopores. The former are based on protein nanopores that have been employed to great success for the real-time sequencing of nucleic acids.^21,22^ The latter offer an alternative based on inorganic materials that could provide higher signal-to-noise ratio (SNR), diameter tunability and improved stability.^1,23,24^

The analysis of small nucleic acids and proteins with solid-state nanopores represents a sensitivity challenge, as the translocation of these small molecules (relative to a pore size of <30 nm diameter) leads to a signal that is difficult to distinguish reliably from the background current.^1,24^ While several approaches have been used to address this challenge, they only partially solve this problem. For example, existing methods rely on using nanopores of few nanometers in diameter embedded in nanometer-thick membranes integrated with custom designed electronics with MHz bandwidth.^11,23^ This approach allows for high SNR and submicrosecond temporal resolution, but it requires access to highly specialized and costly equipment. Alternative approaches rely on the modification of the physical-chemical properties of the solutions used in nanopore experiments. For example, the viscogen glycerol has been added to the electrolyte to reduce the speed of the molecular translocations, but at the expense of a reduced SNR.^25^ Another approach relies on using LiCl as the electrolyte to slow down the translocation of molecules through nanopores; this approach is particularly effective for nucleic acids but does not increase the SNR.^26^ Salt gradients can also be used to improve the translocation frequency across a nanopore, but this affects neither the speed nor the current magnitude of the single molecule events.^27^ Alternatively, the nanopore surface can be chemically modified to slow down the translocation of analytes,^28−32^ but this method is difficult to generalize as it needs to be tailored for each analyte.

We recently reported that including poly(ethylene) glycol (PEG) at a concentration of 50% (w/v) in the bath solution results in a pronounced increase (up to 6-fold) in the SNR facilitating the detection of DNA, globular, filamentous proteins,^33^ and DNA origami.^34^ Here, we build on these observations to present single-molecule nanopore sensing based on the interactions between PEG and the supporting electrolyte. We determine how the electrolyte employed modifies the translocation dynamics of a model analyte (4.8 kb double-stranded DNA (dsDNA)), as characterized by the translocation event current peak magnitude and the dwell time. Our results indicate that only the physical interaction of the electrolyte with PEG in the bath governs the translocation dynamics of the analytes, and that this is independent of the composition of the solution used to fill the nanopipette and dilute the translocating molecules. We investigated the translocation dynamics of a model analyte (4.8 kbp linearized DNA) with a range of electrolytes and discovered a correlation of the single-molecule signals with the lattice energy of the electrolyte, a physical property that has been used to approximate the affinity of an ion to PEG. These experiments allowed us to identify CsBr, a salt seldomly used in nanopore sensing, as the optimal supporting electrolyte to complement the polymer–electrolyte nanopore system. This polymer–electrolyte system allowed us to profile, under physiologically relevant conditions, RNA fragments from the Chikungunya virus (CHIKV) genome and to probe and differentiate distinct RNA conformations of a 1987 nt fragment of the genome with known, distinct, structure. The results allow the conformational analysis of RNA (and other macromolecules) under native conditions using a solid-state nanopore with high sensitivity, including fragments as small as 318 nt long.

## Results and Discussion

**Figure 1:**
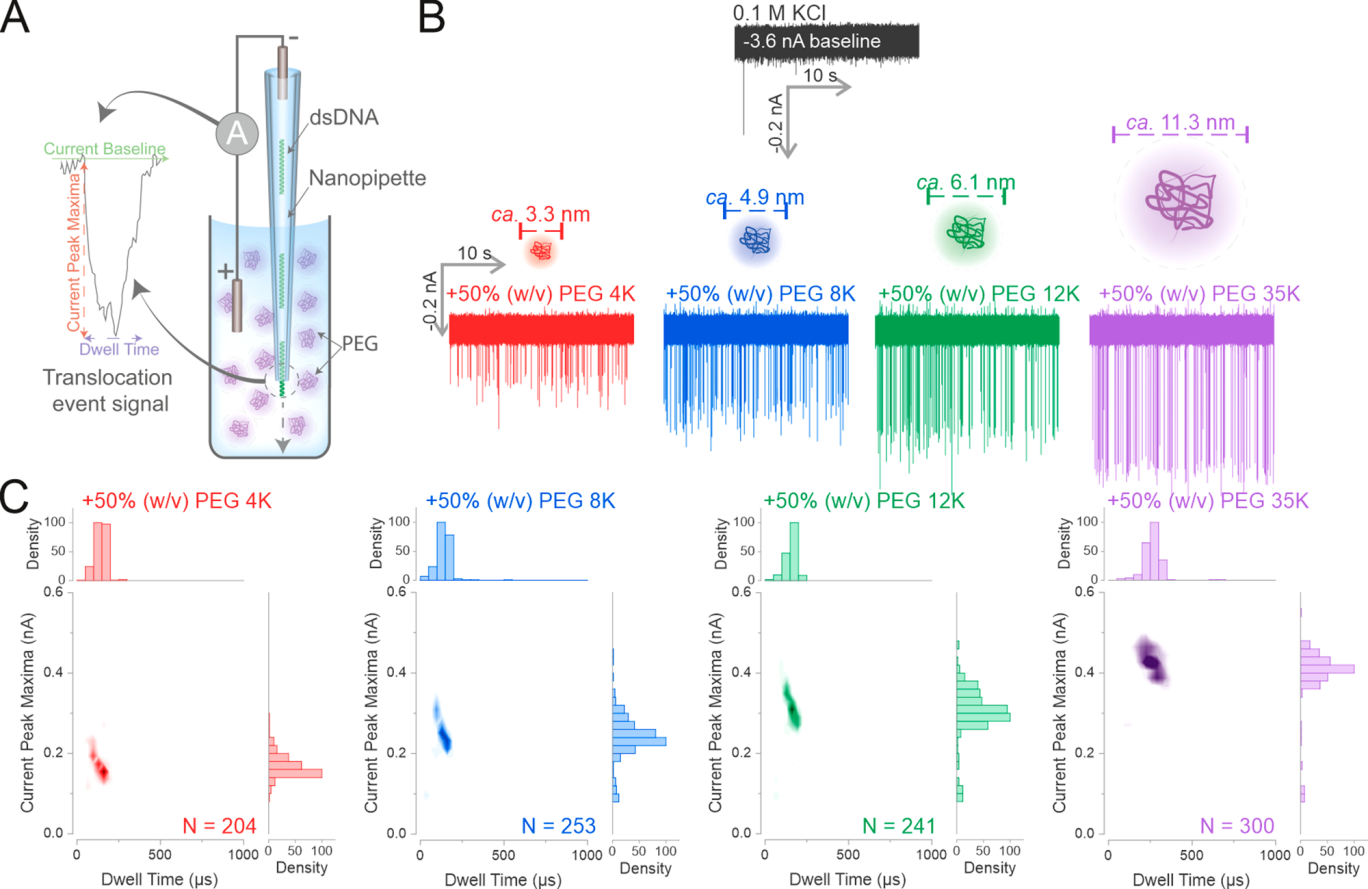
Increasing the MW of PEG enhances the detection of dsDNA by increasing the SNR. (A) Schematic of the polymer–electrolyte solid-state nanopore setup. The dsDNA is diluted in 0.1 M KCl to 0.3 nM and used to fill the nanopipette. The nanopipette is then immersed into a 50% (w/v) PEG bath containing 0.1 M electrolyte and a negative voltage is applied to drive the translocation of the dsDNA. Each translocation of the dsDNA generates a conductive translocation event signal with two main characteristics: the current peak maxima (the change of current level from the current baseline) and the dwell time (the time it requires for the current to return to baseline). (B) The nanopipette was filled with 0.3 nM of a 4.8 kbp dsDNA diluted in 0.1 M KCl, and immersed into a 0.1 M KCl bath. A voltage of −500 mV was appliedto drive the dsDNA translocations. The same procedure was repeated in 0.1 M KCl baths containing PEGs with different molecular weights as stated. The calculated hydrodynamic diameters for the different molecular weight PEGs are shown.^51^ (C) The recorded translocation events in the KCl PEG bath of (B) were used to generate density scatter plots and histograms. Four independent nanopore experiments were performed. *N* is the number of events detected.

In this study, we used a nanopipette as a model solid-state nanopore.^1,2^ Nanopipettes with a diameter of *ca*. 25 nm (Supporting Figure 1) were filled with a solution of 0.3 nM dsDNA (4.8 kbp; Supporting Figure 2) diluted in 0.1 M KCl. The nanopipette was immersed into a 0.1 M KCl bath solution containing 50% (w/v) PEG with a range of different molecular weights (MW) (see Supporting Information for details on the preparation of the bath solutions). The concentration of 50% (w/v) PEG was chosen and kept constant across all experiments, as we previously demonstrated that this provides the highest SNR.^33^ Two Ag/AgCl electrodes, one inside the nanopipette, the other immersed into the bath electrolyte were used to apply the voltage and measure the current. The translocation of a single dsDNA molecule from inside the nanopipette to the bath electrolyte leads to a current enhancing peak (*i.e.*, the dsDNA translocation temporarily increases the measured current), and each peak is a single-molecule translocation event^3,4^ (Figure 1A). Each translocation event can be described by two main parameters: the current peak maxima (the amplitude of the event) and the dwell time (the width of the event).

We observed that increasing the MW of PEG led to an increase in the current peak magnitude of the single-molecule translocation events (Figure 1B). The effect of the PEG size can be seen clearly in the density scatter plots of the translocation events (Figure 1C), where the population of events exhibited higher peak magnitudes and longer dwell times with increased PEG molecular weight (Figure 1C and Supporting Figure 3A,B). The mean current peak maxima of the dsDNA increased from 0.17 ± 0.02 nA in PEG 4K to 0.40 ± 0.01 nA in PEG 35K, while the dwell time increased from 130 ± 10 μs in PEG 4K to 300 ± 30 μs in PEG 35K, a nearly 2-fold increase (Supporting Figure 3A,B). We performed control experiments to ensure that the translocation signals detected were due to the migration of dsDNA from nanopipette to the bath and not to the translocation of PEG itself, as this has been reported for biological nanopores and nanopipettes.^35−43^ We did not observe any translocation events when the nanopipette was filled only with 0.1 M KCl and immersed into the KCl PEG 35K bath (Supporting Figure 4). PEG 35K generated the most significant enhancement in the SNR for dsDNA and it is used throughout this study unless otherwise stated.

Due to the inherent high frequency dielectric and capacitive noise,^24^ most solid-state nanopore translocation experiments need to apply a low-pass filter (typically at 10 kHz and above) to filter the high frequency noise such that the translocation event signals can be distinguished from the current baseline, but at the cost of distorting the translocation event signals.^44,45^ Here, we examined the translocation of a 4.8 kbp dsDNA at −500 mV into the PEG bath with different low-pass filter settings. Translocation events could still be observed even when the low-pass filter was bypassed (Supporting Figure 5). In contrast, when we used PEG 4K, no translocation events could be observed without a low-pass filter, indicating that the translocation events were concealed by the high frequency noise (Supporting Figure 5). We acquired the ion current data at 2 μs intervals (500 kHz) and translocation events of the 4.8 kbp dsDNA could be detected under an applied voltage as low as −300 mV (Supporting Figure 6).

To explore how the PEG electrolyte bath increases the SNR, we measured the shear viscosity and the conductivity of the PEG electrolyte (Supporting Figure 3C,D) to investigate how the properties of the electrolyte bath may affect the SNR. A solution of PEG 35K in 0.1 M KCl had a viscosity of nearly 10 Pa·s, which is approximately 100× higher than PEG 4K and 10,000× more viscous than a 0.1 M KCl solution in absence of PEG. In contrast, the conductivities of all 0.1 M KCl PEG solutions were approximately 1.5 mS/cm, regardless of the MW of PEG, a ∼10× reduction compared to the conductivity of 12 mS/cm in 0.1 M KCl. The shear viscosity and the conductivity values measured here are in agreement with those reported in the literatures.^46,47^ It is interesting to observe that the current peak maxima did not increase by 10× between the PEG 12K and 35K solution despite a ∼10× difference in solution viscosity, which indicates that viscosity alone cannot fully explain the observed enhancement. Furthermore, these results agree with our previous observations that the viscogen glycerol does not lead to any signal enhancement.^33^ However, it is plausible that the prolonged translocation dwell time in PEG 35K could be related to the increase in the solution viscosity, as Fologea et al. observed this effect in a solid-state nanopore system.^25^ Since the excluded volume effect due to the addition of PEG (*i.e.*, the macromolecular crowding effect) also increases as PEG MW is increased,^48−50^ macromolecular crowding may also play a role in addition to the effect of solution viscosity.

We also analyzed the relationship between the molecule count and the applied voltage (Supporting Figure 7). This relationship can be either barrier-limited or diffusion-limited and it reflects how the molecules are captured during the translocation process.^9,13,16^ The results showed an exponential relationship of the number of molecules translocated versus the applied voltage in a 0.1 M KCl solution, suggesting a barrier-limited capturing of the dsDNA. By contrast, in the presence of PEG, the capture of dsDNA follows a linear relationship, suggesting a diffusion-limited regime that is dependent on the magnitude of the applied voltage (Supporting Figure 7).^9,13,16^ The main difference that leads to either the barrier-limited or the diffusion-limited regime is the local ion environment near the nanopore.^9,13,16^ This observation is intriguing, as it implies that the addition of PEG to the bath alone alters the local ion environment near the nanopore and hence the detection mechanism is potentially different from that of previously studied models.^3,4,15,27^ The signal enhancement is likely influenced by an interplay of the physical properties of PEG and its effects on the concentration of ionic species in the nanopore.

**Figure 2:**
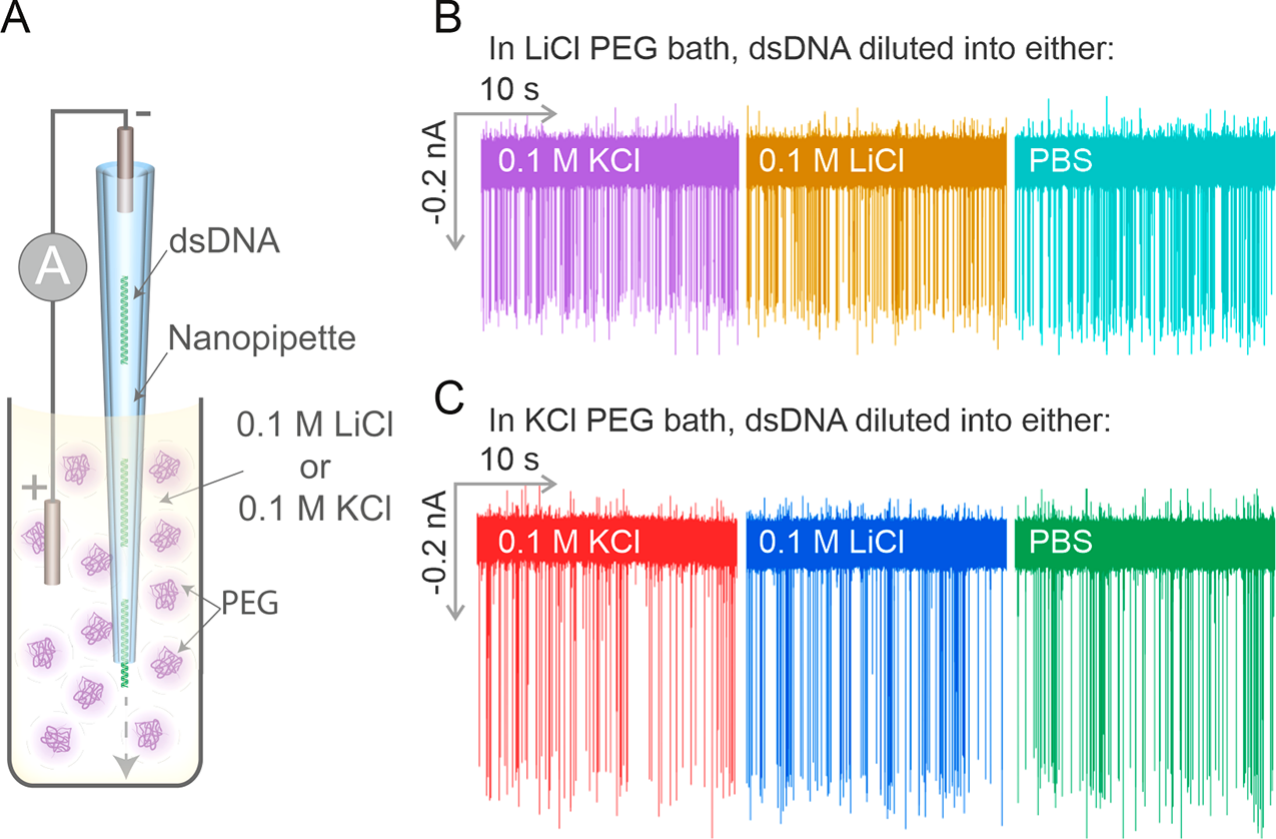
Bath electrolyte controls the characteristics of the translocation event signals. (A) Schematic of the experimental setup. The dsDNA filled nanopipette was immersed into either a 0.1 M LiCl PEG 35K or a 0.1 M KCl PEG 35K bath. (B) The dsDNA was diluted into either 0.1 M KCl, 0.1 M LiCl, or PBS and immersed into a 0.1 M LiCl PEG bath to perform the translocation experiments. (C) The dsDNA was diluted in either 0.1 M KCl, 0.1 M LiCl, or PBS and was immersed into a 0.1 M KCl PEG 35K bath to perform the translocation experiments. Five independent nanopore experiments were performed.

We also studied how the physicochemical properties of the electrolyte could also influence the characteristics of the translocation events. The importance of the DNA counterion cloud in determining the amplitude of the single-molecule events is well established. For example, in 0.1 M KCl, the translocation of DNA through a nanopipette elicits a temporary current enhancement rather than a reduction, due to counterion charge screening on the DNA molecule.^52,53^ LiCl is also commonly used in nanopore detection of nucleic acids because of its ability to slow down the velocity of translocations.^26,54^ Therefore, we tested whether a combination of PEG and LiCl could enhance the SNR and reduce the translocation speed of dsDNA (Figure 2A). We observed that LiCl did indeed reduce the translocation speed of dsDNA, but with a reduced SNR compared to KCl (Supporting Figure 8).

Interestingly, we observed that only the bath electrolyte influences the magnitude and dwell time of the single-molecule events, while the electrolyte/buffer within the nanopipette, where the analytes are placed, plays a negligible role (Figure 2B,C). We demonstrated this observation by diluting dsDNA into either 0.1 M KCl, 0.1 M LiCl, or PBS and performed translocation experiments into a 50% PEG 35K bath containing either 0.1 M KCl (Figure 2B) or 0.1 M LiCl (Figure 2C). As both the current trace and population scatter data show, the events all had similar current event magnitudes irrespective of whether KCl, LiCl, or PBS were used to dilute the dsDNA, whereas the current peak amplitude changed when the PEG bath contained different salt species. This is significant because different analytes require specific buffer conditions and ionic strength to maintain their integrity, *e.g*., DNA origami nanostructure and ribosomes both require the presence of Mg^2+^ ions to stabilize their conformation^55,56^ and protein structure can become unstable at high salt concentrations.^57^ This decoupled relationship between the buffer where the analyte is dissolved and the bath electrolyte will be used later in the manuscript to probe the conformation of a viral RNA genome under physiological conditions.^58−60^

The increase in dwell time when LiCl is used for DNA molecule translocation experiments has been reported before, and this effect was explained by the stronger binding affinity of Li^+^ to the DNA backbone compared with other cations.^26,54^ However, this scenario is unlikely to occur in our experimental setup, as when the dsDNA solution was diluted in either the presence or absence of LiCl, identical population scatters were produced in the PEG 35K KCl and the PEG 35K LiCl electrolyte baths (Supporting Figure 8). This indicates that the counterion shielding on the negatively charged phosphate backbone of the DNA appears to play a negligible role in determining the shape and duration of the single-molecule events in our experiments. These results, therefore, suggest that an interaction between Li^+^ and the PEG molecules in the electrolyte bath is likely to drive the signal enhancement. We therefore hypothesized that a cooperative effect between the salt species and PEG in the electrolyte bath modulates the signal enhancement and may also affect the translocation event signals.

**Figure 3:**
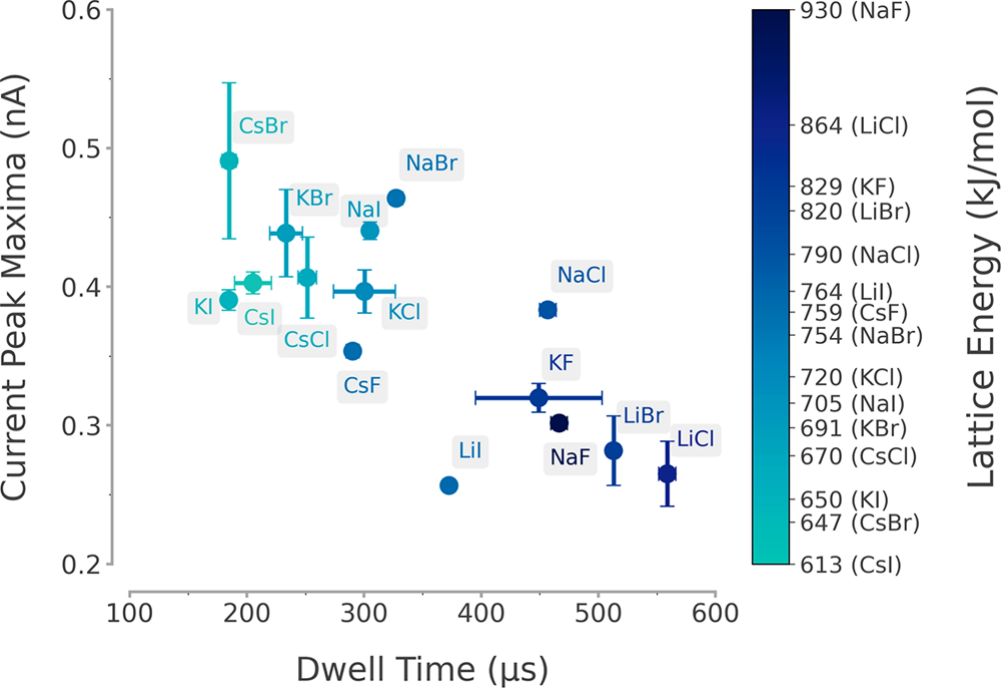
Influence of different alkali metal halide salts on the translocation event signals of 4.8 kb linearized dsDNA. The dsDNA was diluted to 0.3 nM in 0.1 M KCl, and this solution was used to fill the nanopipettes. Different 0.1 M alkali metal halides were used to generate the PEG 35K bath; these were LiCl, LiBr, LiI, NaF, NaCl, NaBr, NaI, KF, KCl, KBr, KI, CsF, CsCl, CsBr, and CsI. The KCl diluted dsDNA nanopipettes were immersed into the different metal halide PEG 35K baths, and −500 mV was applied to drive the dsDNA translocations. The plot displays the mean current peak maxima and the dwell time, and each salt is color coded with its associated lattice energy. Three independent nanopore experiments were performed for each salt condition.

To test our hypothesis, we prepared a range of alkali metal halide solutions that were each dissolved at a concentration of 0.1 M in 50% (w/v) PEG 35K. Linearized 4.8 kb DNA was diluted in 0.1 M KCl and translocation experiments were carried out at −500 mV (Supporting Figures 9 and 10). The results obtained indicate that the nature of the electrolyte affects both the magnitude of the current and dwell time of the single-molecule translocation events (Figure 3). We found that CsBr caused the greatest amplification of the current peak maxima, although it resulted in the shortest dwell time among the salts tested. Conversely, LiCl generated the largest increase in the average dwell time but had the smallest enhancement of the current magnitude.

We observed a trend in which salts of higher molar masses, *e.g.*, CsBr, KI, CsI, had stronger amplification of current peak maxima, while salts with lower masses such as LiCl, LiBr, and NaF generally showed longer dwell times (Figure 3). The decrease in the dwell time and increase in the current peak maxima was associated with an increase of the atomic number of the anions (*e.g*., from KF to KCl to KBr and KI, Supporting Figure 10), which could be due to the differences in mobility between the cation and the anion.^61^ We also noticed that the Li^+^ and Na^+^ solutions had different effects on the translocation events compared to the K^+^ and Cs^+^ ions, based on the population scatters generated (*e.g.*, LiCl and NaCl vs KCl and CsCl, Supporting Figures 9 and 10). These observations hinted that the physical properties of the salt could be the cause of the observed differences in the translocation event characteristics between different salt species.

The use of PEG as a model analyte for biological nanopores has been well documented,^35−40^ and several studies have demonstrated the ability of PEG to interact with cations in solution.^41,42^ These studies utilized the biological nanopore α-hemolysin to detect the translocation of PEG and showed that the PEG molecules were neutral when a Li^+^ containing electrolyte was used.^41,42^ In contrast, PEGs were positively charged when other alkali metal halides were used, including the cations Na^+^, K^+^, Rb^+^, and Cs^+^. In 1982, Papke et al. studied the thermodynamic properties of the PEG–electrolyte interactions with sodium containing electrolytes and showed that the lattice energy of these electrolytes could be used to estimate their likelihood to interact with PEG.^62^ Lattice energy is the energy required to separate a mole of an ionic solid into gaseous ions, and it is inversely proportional to the ionic compound’s molar mass. We observed that the average current peak maxima were inversely proportional to the lattice energy, while the average dwell time was proportional to it (Figure 3, Supporting Figure 11, Supporting Table 1). Overall, our results show that the nature of the electrolyte is a major determinant for the dwell time and current maxima of dsDNA translocation. These differences can be explained by the interaction between the salts and PEG, as approximated by the use of lattice energy,^62^ indicating that the cooperative interaction between the electrolyte and PEG inside the bath is a key for the observed signal enhancement.

We tested PEGs of different sizes and the experimental results showed that increasing the molecular weight of the PEG is associated with a stronger current enhancement (Figure 1), which could be due to the increased crowding effect caused by increasing the size of the PEG.^48,49^ Although PEG can be used as a macromolecular crowder, PEG is also known for its ability to chelate cations,^63^ a property that has been extensively studied in the field of Li-ion batteries,^64^ and cation chelation could explain some of our observations. Indeed, when an electric field is applied in a solution containing PEG, the cation migration is hindered by the interactions with the polymer itself, thus causing a reduction in the cation mobility.^64−66^ To further understand the mobilities of different cations in the PEG electrolyte, Zhang et al. employed molecular dynamics (MD) simulations, validated with quasi-elastic neutron scattering, to simulate the mobilities of Li^+^, K^+^, and Na^+^ cations.^67^ The study showed that cation K^+^ is chelated by PEG and spent nearly 5 ns interacting with the PEG chain, in contrast to Li^+^, which is more mobile and spent less than 1 ns interacting with the PEG chain.^67^ Herein, we propose an explanation that the observed differences in the translocation event signals between the Li^+^, Na^+^, K^+^, and Cs^+^ bath solutions could be due to the differences in how strongly these cations interact with the ethylene oxide group of PEG.

To further explore the SNR enhancement achieved by using CsBr, we studied a shorter, 500 bp fragment of dsDNA (Supporting Figure 12) to compare the effects of CsBr, KCl, and LiCl in the PEG electrolyte bath. The detection of short dsDNA (<1000 bp) can be challenging with glass solid-state nanopores because the SNR is often very small due to a poor analyte to pore ratio^68^ and the translocation of short DNAs through the nanopore is too fast to be detected^28,29,69^ even with state-of-the-art electronics.^70^ In order to ensure that the signals obtained were not due to slight deviations of nanopore size during fabrication, the same nanopipette filled with the 500 bp dsDNA was used for translocation experiments in all three salt PEG baths. Translocation events could be detected for all three salt PEG baths, but CsBr yielded the highest molecule count within 30 s of the translocation experiment, leading to *ca*. 7× higher molecule count compared to KCl or LiCl (Supporting Figure 12).

**Figure 4:**
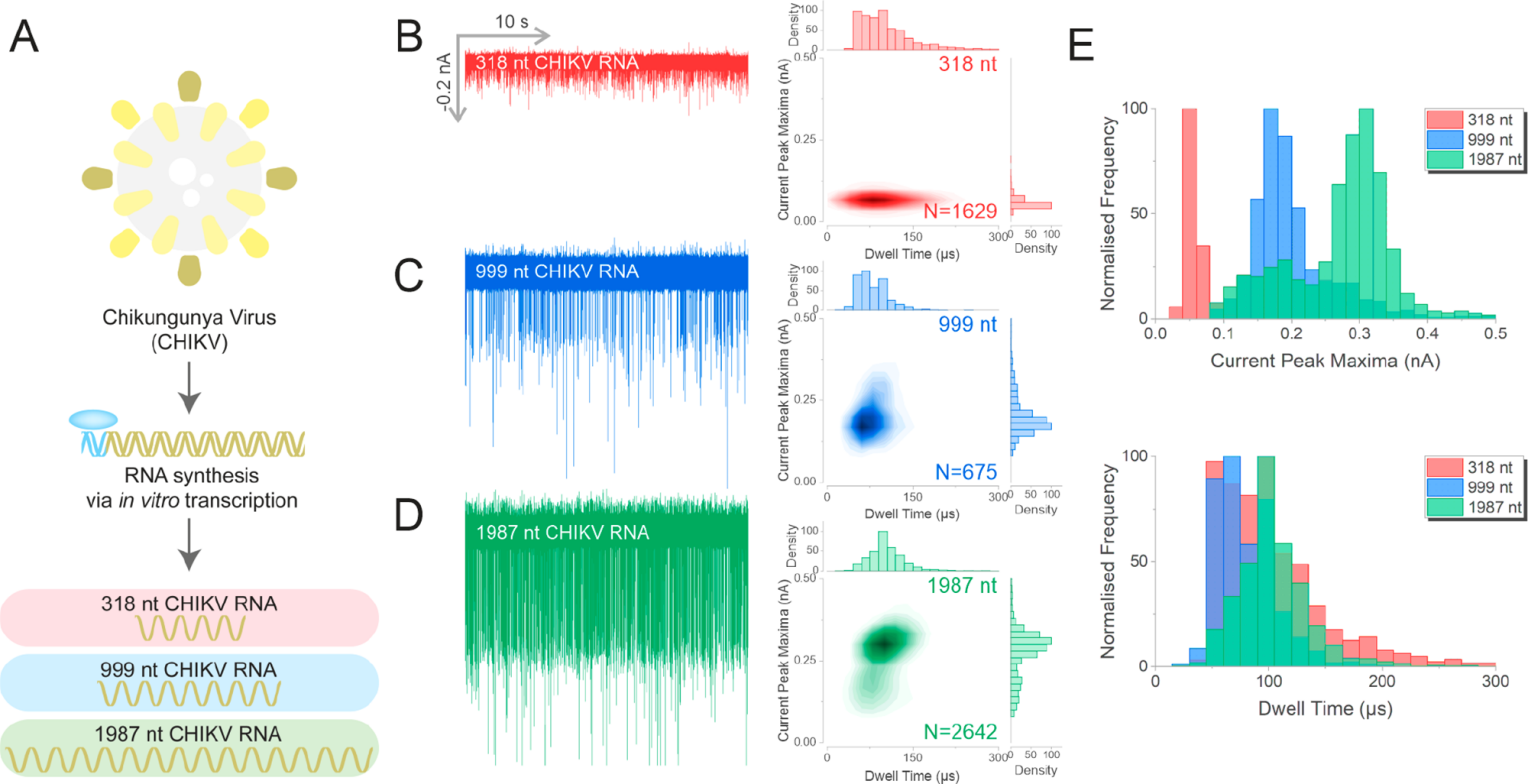
Nanopore analysis of different lengths of CHIKV RNA using a CsBr PEG bath. (A) Schematic of the generation of Chikungunya virus (CHKIV) RNAs of different lengths by T7 RNA polymerase. Three different lengths of CHIKV RNA were generated: 318 nt (red), 999 nt (blue), and 1987 nt (green). These RNAs were diluted to 30 pM in RNA folding buffer (111 mM HEPES, 6.67 mM MgCl_2_, 111 mM NaCl at pH 8.0) prior to analysis. The translocation traces of 318 nt (B), 999 nt (C), and 1987 nt (D), and the associated population scatters are shown. (E) Histograms of the current peak maxima and the dwell time for the three RNA fragments. Three independent nanopore experiments were performed for each RNA sample. *N* is the number of events detected.

Building upon these findings, we analyzed RNA fragments of the CHIKV genome with defined length and conformation at physiologically relevant conditions with the polymer–electrolyte nanopore system. CHIKV is a re-emerging, pathogenic alphavirus transmitted to humans by mosquitoes.^71^ Here, the CHIKV infectious clone was used as the cDNA template for RNA generation by *in vitro* transcription (Supporting Information). Using different primer combinations, we generated cDNA templates of three different lengths: 318, 999, and 1987 nt for the transcription of viral RNA (Figure 4A, Supporting Figure 13). The resultant RNA fragments were diluted to a final concentration of 30 pM in the buffer containing 0.11 M NaCl. The translocation of the RNA fragments into the CsBr-PEG bath generated well resolved single-molecule events for all the RNA lengths investigated (Figure 4B–D), which was in marked contrast to the translocation of these RNA fragments into an electrolyte bath in the absence of PEG (Supporting Figure 14). Not only could we detect RNA of 318 nt in length, but we were also able to distinguish between the RNAs of different lengths. From overlays of the normalized histograms of the current peak maxima for the three samples, it is evident that our system could resolve the three fragments with an average current peak maximum for the 318, 999, and 1987 nt fragments centered at 0.05, 0.17, and 0.31 nA, respectively. In contrast, our system could not discriminate the length of the RNA fragments based on the translocation dwell times, which were independent of the fragment size and all centered at around 100 μs (Figure 4E). Furthermore, nanopore measurements carried out with the 1987 nt fragments revealed the presence of two populations, a major one centered at ∼0.30 nA and a minor population centered at ∼0.20 nA, which could indicate the presence of distinct RNA conformations.

Single-stranded RNA molecules can fold into an ensemble of conformations, stabilized predominantly by Watson–Crick base pairing, with various compactness and stability landscapes regulated by the impact of its nucleotide sequence on the free energy of base pairing and stacking energies in the context of local ionic conditions and temperature.^60,72^ RNA strands are known to adopt different conformations during catalyzed transcription using RNA polymerase *in vitro*.^60,72^ During the early stages of transcription, the partly synthesized RNA strands will start to fold, as the folding of RNA occurs faster than the transcription rate of the RNA polymerase, this conformation is known as the co-transcriptionally folded conformation.^73^ The co-transcriptionally folded RNA may not necessarily represent the most thermodynamically stable conformation, referred to as the native state.^74^ However, the native thermodynamically stable structure can be modeled *in vitro* by melting the RNA transcripts and refolding them under physiologically relevant temperatures and ionic conditions into the natively refolded conformation.^60,72^

The analysis of RNA conformations is typically achieved by either chemical probing with selective 2′ hydroxyl acylation analyzed by primer extension (SHAPE) method^72,75^ or biophysical technique like single-molecule Förster resonance energy transfer (smFRET)^76^ or cryogenic electron microscopy.^77^ These methods are very informative, but sample preparations can be complicated and the measurements and analysis are often time-consuming. Solid-state nanopore based experiments are easy to prepare and quick to perform, and data analysis is less time-consuming; they have been used previously to detect RNA conformations.^78−84^ However, all these studies were performed at salt concentrations (commonly between 0.3 and 1 M KCl) significantly higher than physiologically relevant levels, and hence it is likely that the conformations of the RNAs will differ from those formed under physiological ionic strength conditions.^60^ Our polymer–electrolyte nanopore system uncouples the solution used to dilute the analyte from the bath solution and it provides an opportunity to study RNAs in electrolyte which facilitates the refolding of RNA into native structures under physiologically relevant conditions.

**Figure 5:**
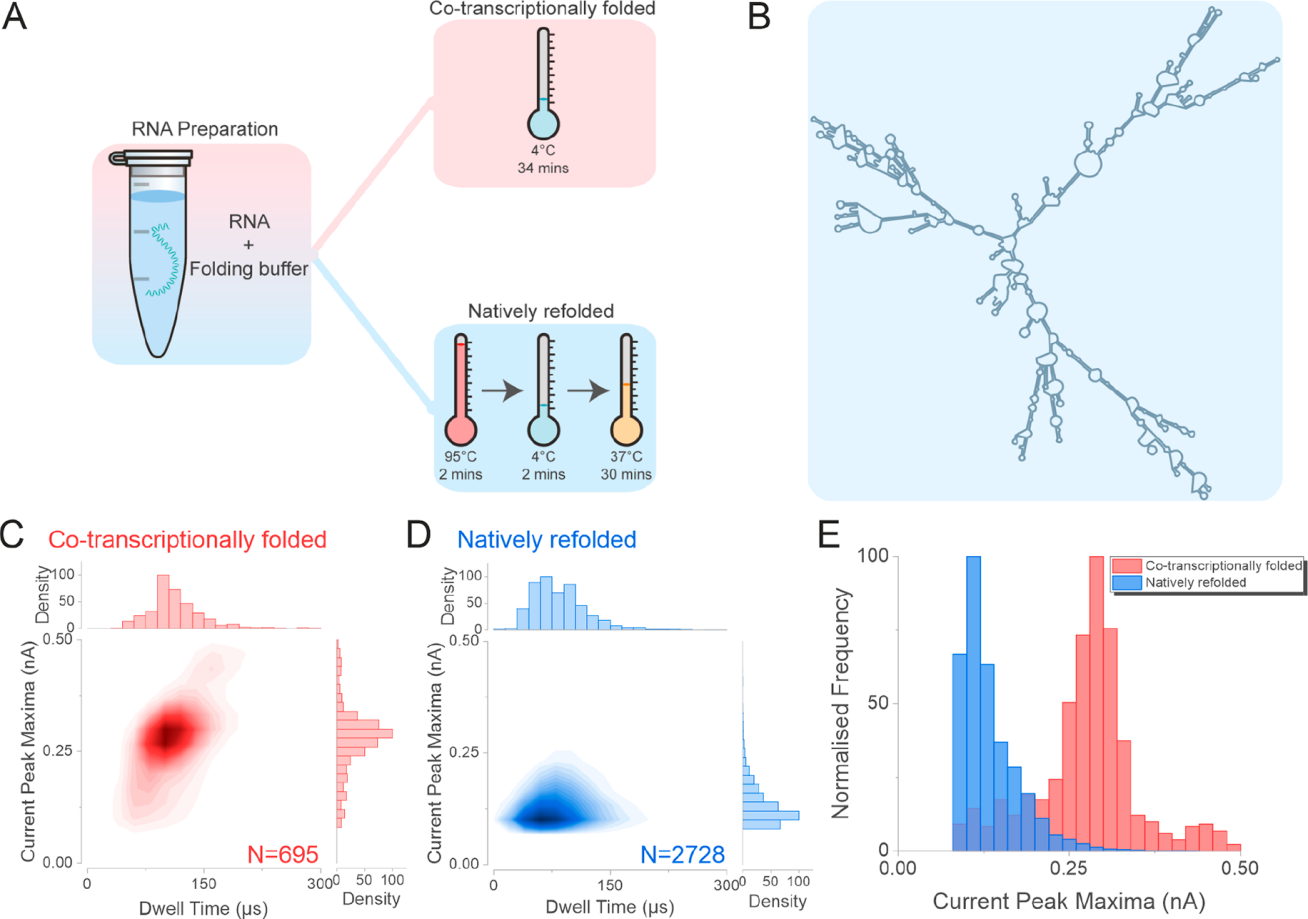
Nanopore analysis of the co-transcriptionally folded and natively refolded CHIKV RNA genome. (A) The 1987 nt CHIKV RNA was transcribed *in vitro* and then either incubated at 4 °C (co-transcriptionally folded, red panel) or subject to the refolding procedure to enable it to adopt a stable (native) conformation (natively refolded, blue panel). The RNA was incubated at 95 °C for 2 min followed by 4 °C for 2 min and finally at 37 °C for 30 min. (B) An *in silico* free energy minimization model, showing the predicted secondary structure of the natively refolded 1987 nt RNA fragment. The population scatters of the co-transcriptionally folded RNA (C) and the natively refolded RNA (D). (E) The current peak maxima histogram of the co-transcriptionally folded (Red) and the natively refolded CHIKV RNA (Blue) translocation data. Four independent nanopore experiments were performed for each RNA sample. N is the number of events plotted.

Two samples of CHIKV RNA were prepared. One (non-native) was co-transcriptionally folded. This sample was then folded into a native conformation by heating and cooling, forming a structure that has previously been characterized structurally and biochemically^85^ (Figure 5A). Here, we used the CHIKV 1987 nt RNA fragment as analyte and employed an *in silico* free energy minimization algorithm to predict the natively refolded CHIKV 1987 nt RNA^86^ (Figure 5B). To test the differences between co-transcriptionally folded and natively refolded RNA, the same 1987 nt RNA fragment was either incubated at 4 °C (co-transcriptionally folded), or subjected to the refolding procedure by incubating the RNA fragment at 95 °C for 2 min followed by 4 °C for 2 min and then 37 °C for 30 min (Figure 5A). The nanopore analysis of the co-transcriptionally folded RNA generated a cluster of events centered at ∼100 μs and 0.29 nA, in agreement with the data presented in Figure 4, while the refolded RNA generated a cluster of events centered at ∼75 μs and 0.11 nA (Figure 5C, D, Supporting Figure 15). Furthermore, the difference between the two samples was clearly evident from the current peak maxima histogram (Figure 5E), suggesting that the refolded RNA adopted a compact conformation that can be probed by using the experimental procedure described herein.

## Conclusion

In this work, we have demonstrated that the physical properties of a polymer–electrolyte bath modify the translocation dynamics of a model analyte, linearized dsDNA, as characterized by the current peak maxima and the dwell time of the translocation signal. Our results suggest a cooperative effect between the electrolyte and the polymer is responsible for driving the signal enhancement in a solid-state nanopore. The translocation dynamics of the analyte into the polymer–electrolyte bath can be explained after considering the cation binding properties of PEG, and that the strength of the interaction between cations and PEG can be used as an approximator for the observed signal enhancement. We then demonstrated that using CsBr together with a PEG 35K electrolyte bath enables the detection of dsDNA at high temporal resolution without the need for low-pass filters and also permits the detection of dsDNA fragments as short as 500bp. Importantly, we show that the polymer–electrolyte bath alone governs the translocation dynamics of the analyte. Building on these observations, we detected RNA fragments of different lengths that originated from the CHIKV viral genome and probed the physiologically relevant conformation differences between the co-transcriptionally folded and natively refolded RNA. We envision that the polymer–electrolyte solid-state nanopore system described in this work can be used to provide additional data on the conformation of RNA under different buffer conditions, adding nanopore analysis as part of the RNA conformation structure analysis toolbox.

## Methods

The Supporting Information contains detailed methods on the generation of the PEG solutions, detailed dsDNA generation, detailed RNA generation and folding, ionic current traces, and scanning electron microscopy micrographs of the nanopipette used.

### Nanopipette Fabrication

Quartz capillaries of 1.0 mm outer diameter and 0.5 mm inner diameter (QF100-50-7.5; Sutter Instrument) were used to fabricate the nanopipette using the SU-P2000 laser puller (World Precision Instruments). A two-line protocol was used: line 1, HEAT 750/FIL 4/VEL 30/DEL 150/PUL 80, followed by line 2, HEAT 625/FIL 3/VEL 40/DEL 135/PUL 150. The pulling protocol is instrument specific, and there is variation between pullers.

### Ion Current Trace Recording and Analysis

The translocation experiment follows a similar procedure from the previous publication.^33^ For dsDNA analysis, the nanopipettes were all filled with 0.3 nM dsDNA diluted in either 0.1 M KCl (P/4240/60; Fisher Scientific), 0.1 M LiCl (CHE2360; Scientific Laboratory Supplies), or PBS (D8537; Sigma-Aldrich) and fitted with a Ag/AgCl working electrode. For co-transcriptionally folded RNA analysis, the nanopipettes were filled with 1 nM RNA diluted in 111 mM HEPES at pH 8.0, 6 mM MgCl_2_, 111 mM NaCl. For natively folded RNA (Native Refolded) RNA analysis, the nanopipettes were filled with 1 nM refolded RNA diluted in 111 mM HEPES at pH 8.0, 6 mM MgCl_2_, 111 mM NaCl. The nanopipettes were immersed in the electrolyte bath with a Ag/AgCl reference electrode. The ionic current trace was recorded using a MultiClamp 700B patch-clamp amplifier (Molecular Devices) in voltage-clamp mode. The sampling bandwidth of these electronics was approximately 52 kHz.^87^ The signal was filtered using a low-pass filter at 30, 20, or 10 kHz or bypass setting, digitized with a Digidata 1550B at a 100 kHz (10 μs) or 500 kHz (2 μs) sampling rate, and recorded using the software pClamp 10 (Molecular Devices). The current trace was analyzed using a custom written MATLAB script provided by Prof Joshua B. Edel (Imperial College London). For translocation events analysis, the threshold level was defined at least 5 sigma away from the baseline; only events that were above this threshold would be identified as the translocation of the molecule.

## Data Availability

The ionic current trace data associated with this paper are openly available from the University of Leeds data repository at 10.5518/1251.

## Supporting Information Available

Figures including nanopore imaging (SEM, gel electrophoresis), dsDNA and RNA size conformation, physical properties of PEG polymer–electrolyte, and analyte translocation traces and associated population scatter plots, table for data used to generate Figure 3, and detailed methods for electrolyte bath preparation, dsDNA generation, RNA synthesis, quality control and sequences, and scanning electron microscopy (PDF)

## Notes

The preprint version of this work can be found in bioRxiv.^88^

## Abbreviations

DNA: deoxyribonucleic acid
RNA: ribonucleic acid
MHz: megahertz
PBS: phosphate buffered saline
PEG: poly(ethylene) glycol
dsDNA: double stranded DNA
SNR: signal-to-noise ratio
MW: molecular weight
CHIKV: chikungunya virus
