# Supplementary material for: Probing RNA Conformations Using a Polymer–Electrolyte Solid-State Nanopore
Source: ACS Nano. 2022 Oct 24;16(12):20075–85. doi: 10.1021/acsnano.2c08312 (PMC9798860; doi:10.1021/acsnano.2c08312)
Supplement: Supplementary file 1 — nn2c08312_si_001.pdf [file nn2c08312_si_001.pdf]

Supporting Information for

Probing RNA conformations using a polymer electrolyte solid-state nanopore

Chalmers Chau [1,2,3], Fabio Marcuccio [2,3], Dimitrios Soulias [2,3], Martin Andrew Edwards [4], Andrew Tuplin [1], Sheena E. Radford [1], Eric Hewitt [1*], Paolo Actis [2,3*]*

[1] School of Molecular and Cellular Biology and Astbury Centre for Structural Molecular Biology, University of Leeds, Leeds LS2 9JT, UK

[2] School of Electronic and Electrical Engineering and Pollard Institute, University of Leeds, Leeds LS2 9JT, UK

[3] Bragg Centre for Materials Research, University of Leeds, Leeds, UK

[4] Department of Chemistry & Biochemistry, University of Arkansas, Fayetteville, AR, 72701, USA

* Corresponding authors c.c.chau@leeds.ac.uk ; e.w.hewitt@leeds.ac.uk ; p.actis@leeds.ac.uk

Table of Contents

Supporting Figures 3

Supporting Tables 18

Methods 19

Electrolyte bath preparation 19

dsDNA generation 21

RNA synthesis and quality control 22

RNA synthesis related sequence 24

Scanning electron microscopy 27

References 28

Supporting Figures

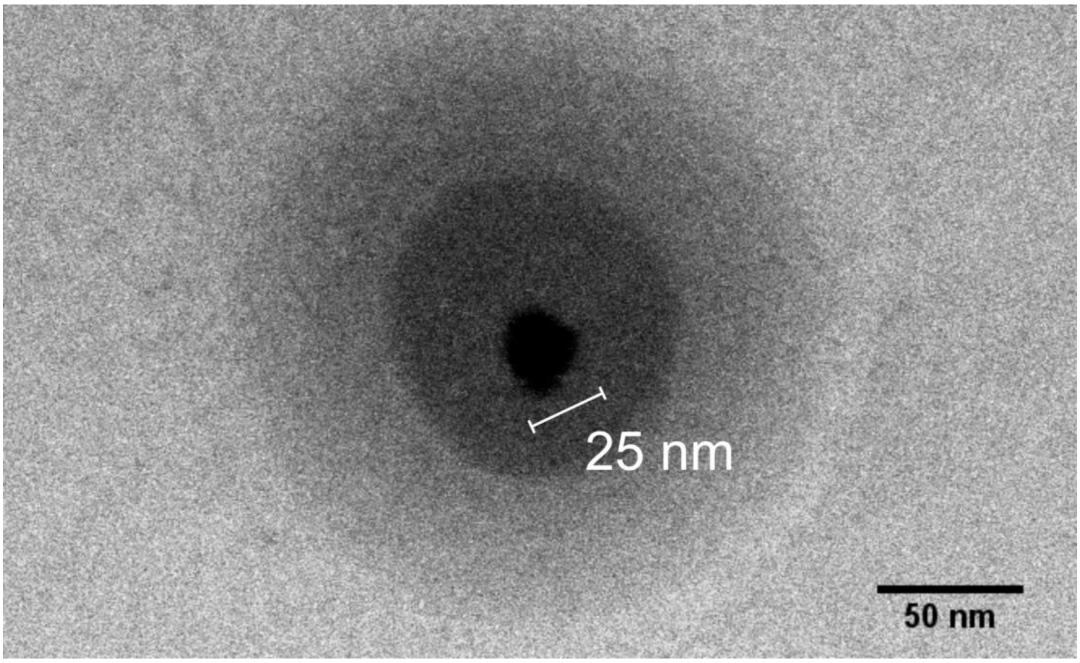

Supporting Figure 1. Scanning electron microscopy image of a nanopipette used in this study. The nanopipettes used in this study had a pore size of approximately 25 nm in diameter.

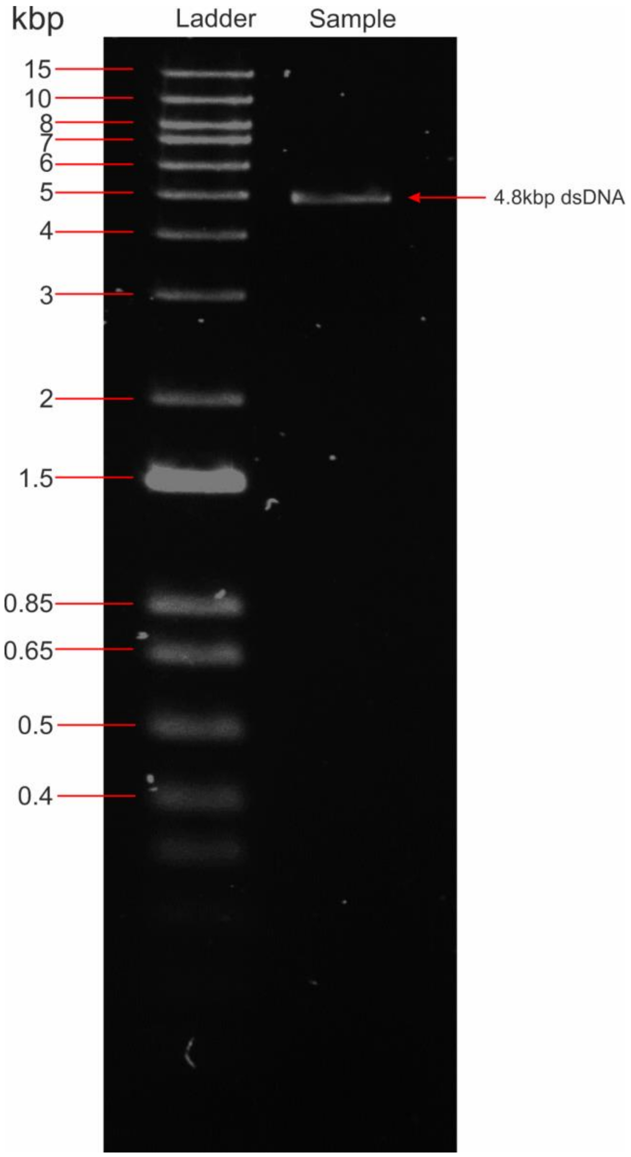

Supporting Figure 2. Gel electrophoresis analysis of the linearized pmCherry-NLS. 1 μg of the linearized plasmid (following the restriction digestion using the enzyme Bam HI) was analysed by agarose gel electrophoresis. A DNA ladder (TrackIt™ 1 kb Plus DNA Ladder (10488085; Thermo Fisher)) was run alongside the digested plasmid sample. The digested plasmid shows a single band of ~5 kbp, which corresponds to the linearized pmCherry-NLS plasmid of *c.a.* 4.8 kbp (4.755 kbp).

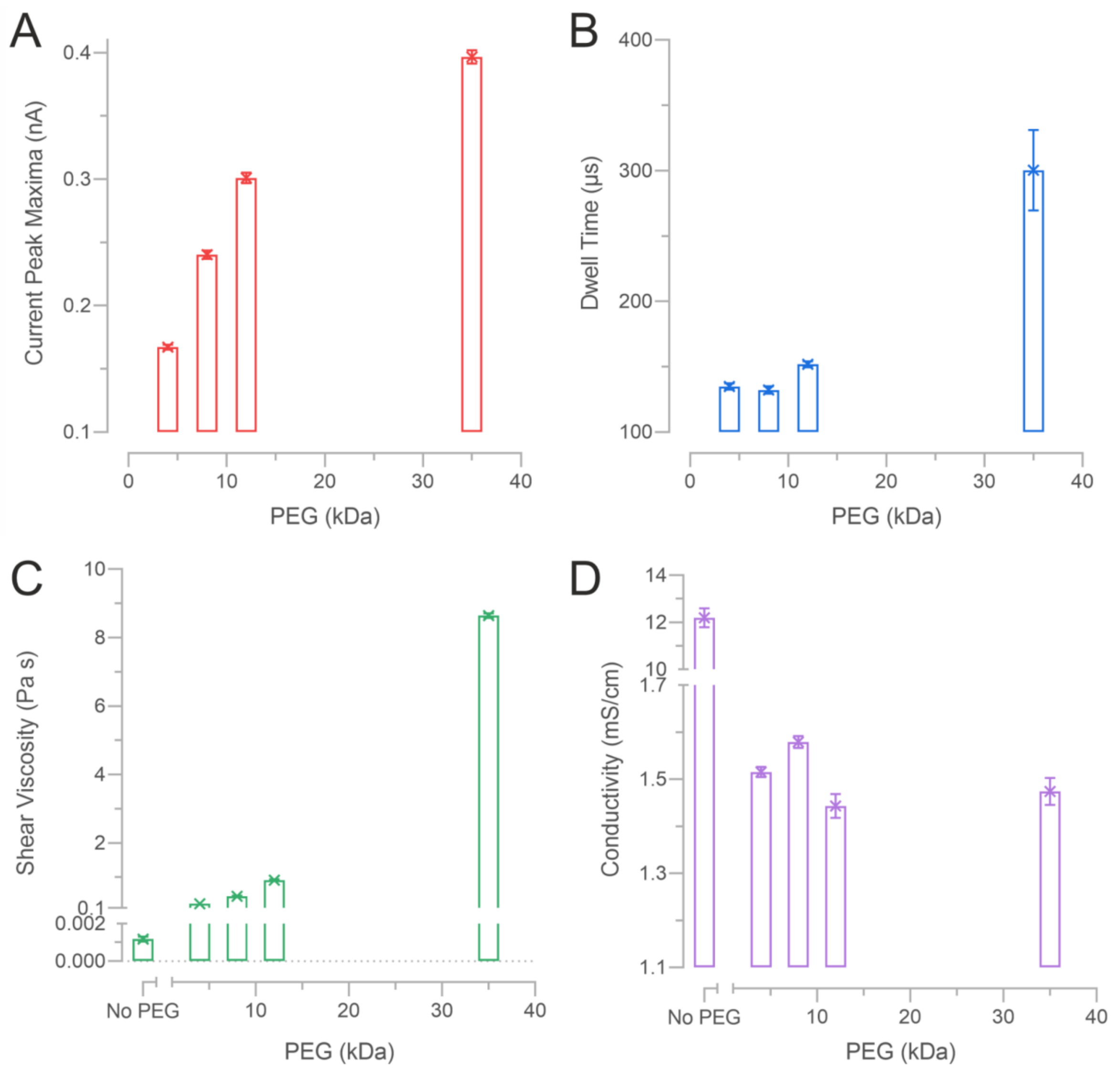

Supporting Figure 3. Summary of the characteristics of dsDNA translocations and the physical properties of baths with different molecular weight PEGs in 0.1M KCl. The average current peak maxima (A) and the dwell time (B) of the translocation events of the 4.8 kbp dsDNA carried out inside the 0.1M KCl electrolyte bath containing different molecular weights of PEG at 50% (w/v). The shear viscosities (C) and the conductivities (D) of the 50% (w/v) PEG 0.1M KCl solutions. Error bars indicate the S.E.M.

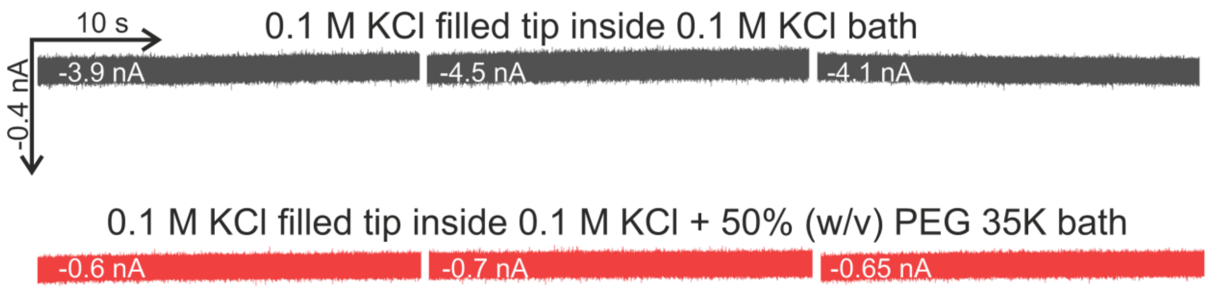

Supporting Figure 4. Current traces for nanopipettes filled with 0.1M KCl in either 0.1M KCl or 0.1M KCl + PEG 35K electrolyte baths. 0.1M KCl was used to fill the nanopipettes and these were then dipped into either a 0.1M KCl or a 0.1M KCl +50% (w/v) PEG 35K bath electrolyte bath, -500 mV was applied for 30 seconds per trace. No translocation events were recorded. The current value indicated inside the traces are the baseline of the current trace. Three traces are shown for each condition. The value superposed to the trace represents the ion current baseline.

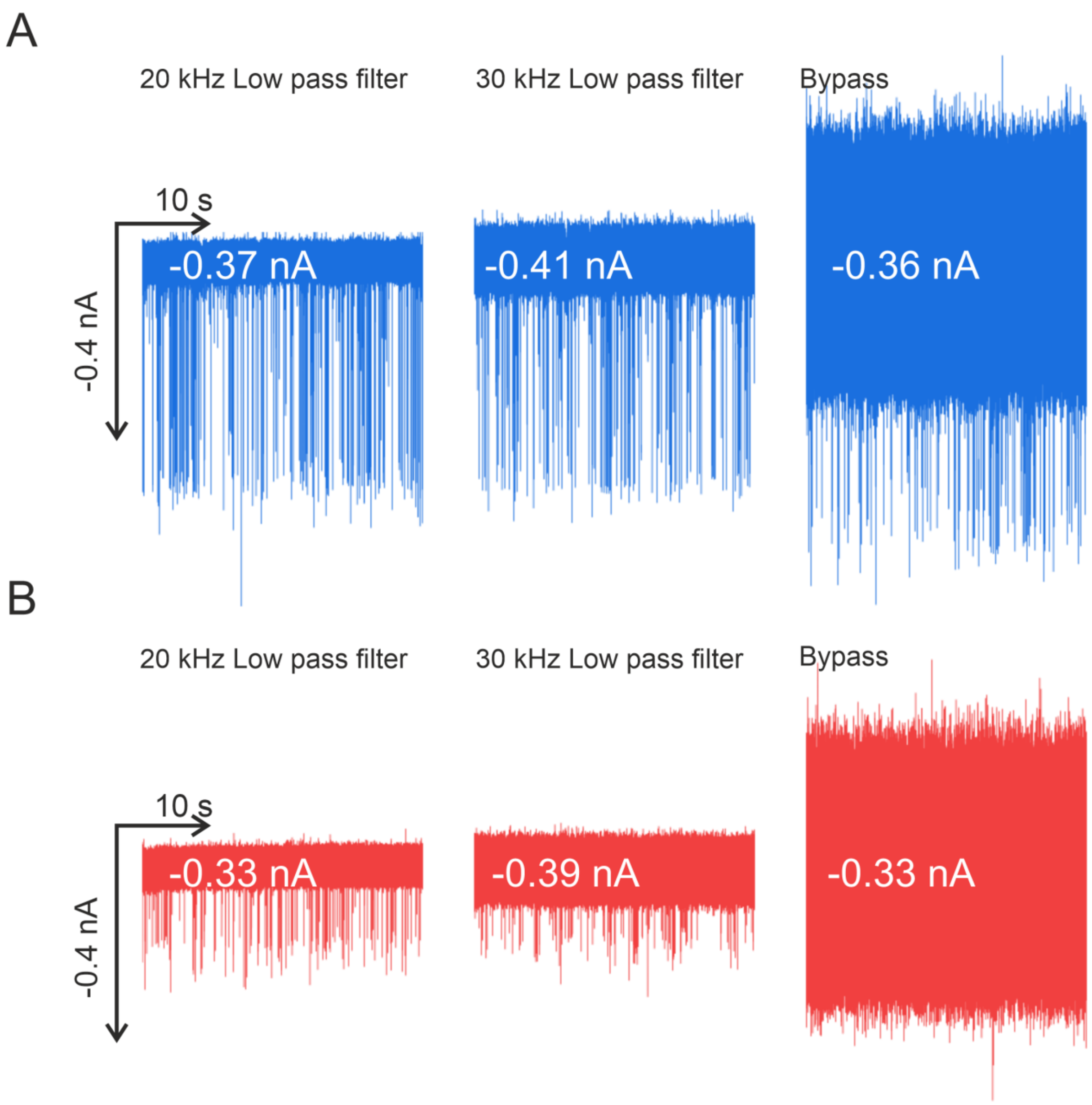

Supporting Figure 5. The translocation of a linearised 4.8 kbp dsDNA into a PEG bath measured with different low-pass filter settings. (A) The translocation of the linearised dsDNA at -500 mV into a 0.1M KCl+50% (w/v) PEG 35K electrolyte bath with either 20, 30 kHz or bypassed low-pass filter. (B) The translocation of the dsDNA at -500 mV into a KCl +50% (w/v) PEG 4K electrolyte bath with either 20, 30 kHz or bypassed low-pass filter. The value superposed to the trace represents the ion current baseline.

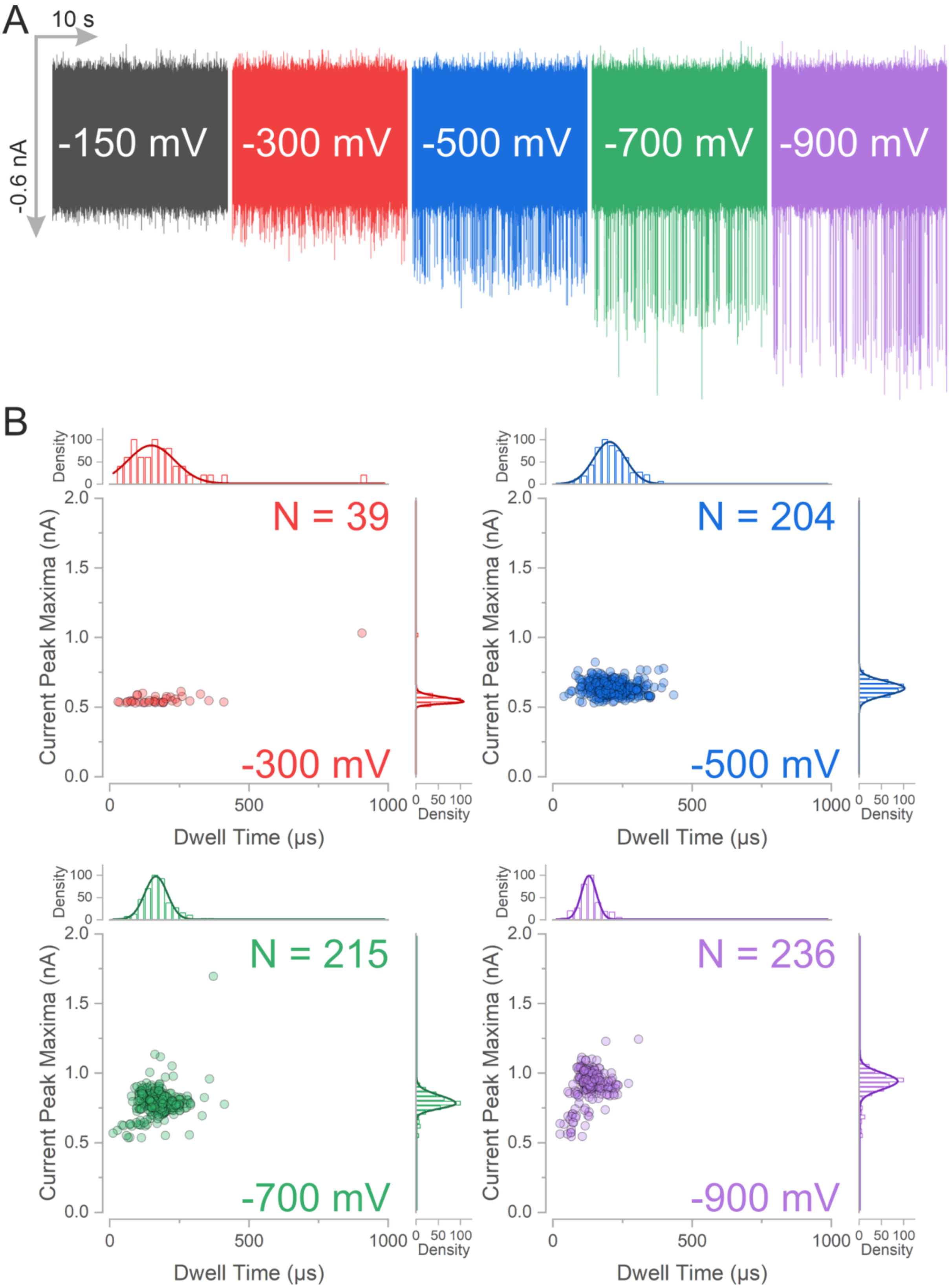

Supporting Figure 6. Voltage dependent translocation of a 4.8 kbp dsDNA into the PEG KCl bath without low-pass filter. (A) Five different voltages from -150 to -900 mV to trigger the translocation of the 4.8 kbp dsDNA into a 0.1M KCl +50% (w/v) PEG 35K electrolyte bath. (B) The population scatter plots for the translocation of the 4.8 kbp dsDNA at each voltage. N indicates the number of translocation events detected.

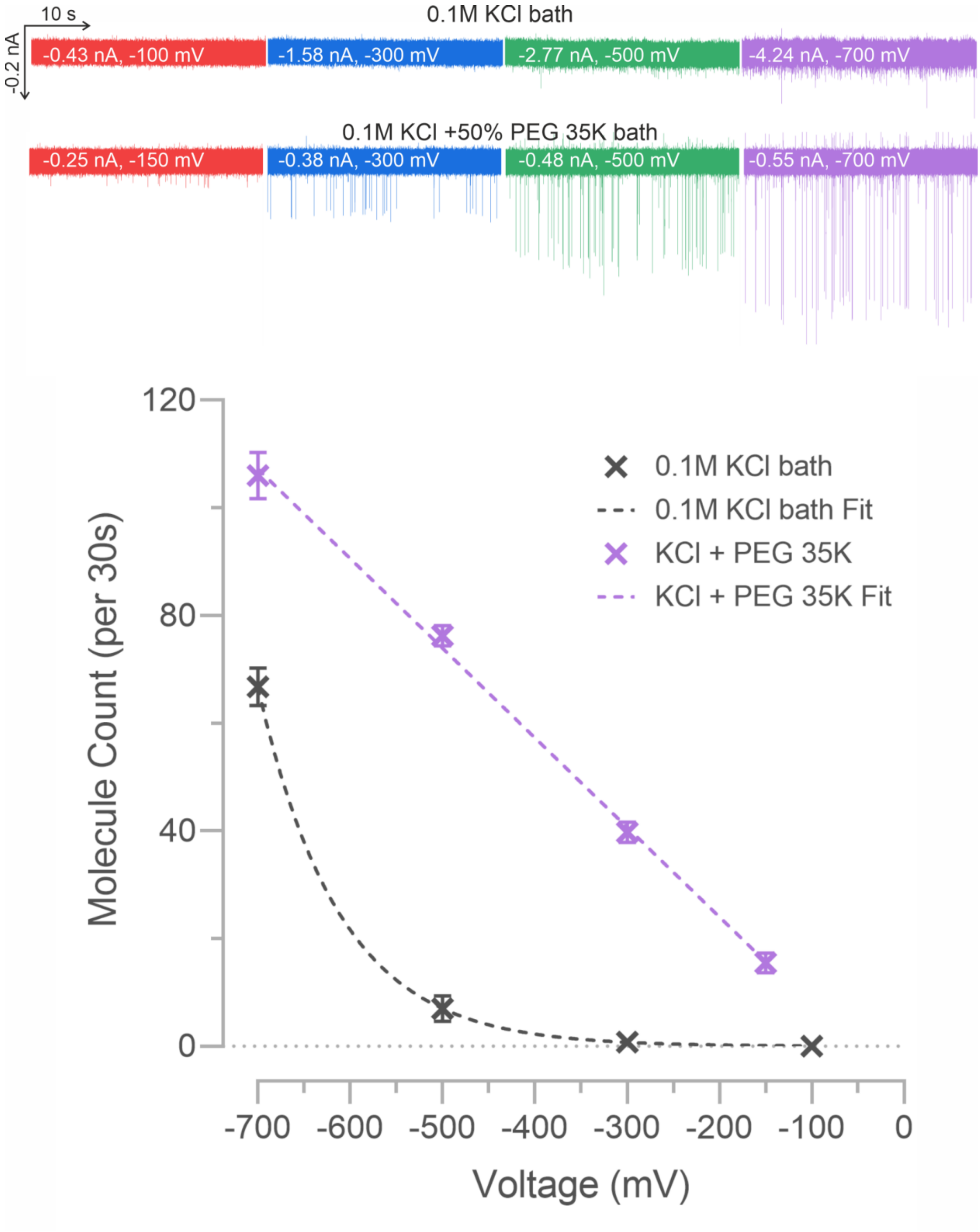

Supporting Figure 7. Voltage dependent translocation of dsDNA. A nanopipette filled with 0.3nM of the 4.8 kbp dsDNA was dipped into either a 0.1 M KCl or a 0.1M KCl +50% (w/v) PEG 35K bath. Four different voltages ranging from -100 to -700 mV were used to cause the dsDNA to translocate from the nanopipette to the bath. The average molecule count from three 30 second current trace is shown for each voltage. The current value indicated inside the traces are the baseline of the current trace. The voltage dependent translocation of the dsDNA inside the 0.1M KCl bath (grey) versus the +50% PEG 35K bath (purple), an exponential fit was performed for the 0.1M KCl data and a linear fit was performed for the PEG 35K data. Error bars indicate the S.E.M, some of the error bars are not shown due to overlapping with the symbol. The value superposed to the trace represents the average values of the ion current baseline. The equations used for fittings are $y=0.02494e^{-0.01127x}$ for 0.1 M KCl bath and y=-0.1664x-9.28 for KCl + PEG 35K Fit.

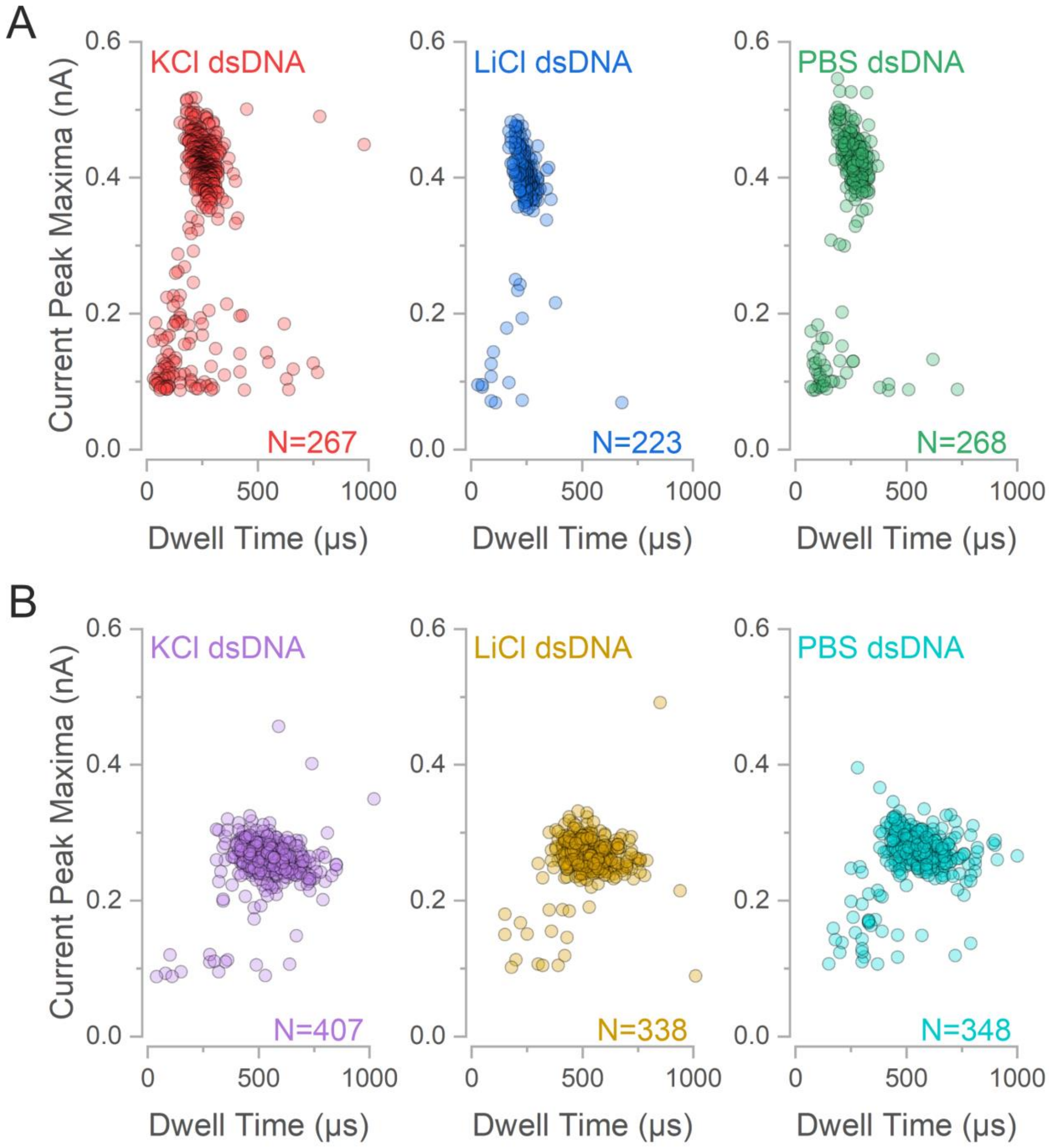

Supporting Figure 8. Population scatter plots for events recorded when dsDNA was diluted into different electrolytes and translocated into either KCl PEG 35K or LiCl PEG 35K electrolyte baths. The 4.8 kbp dsDNA was diluted in either 0.1M KCl, 0.1M LiCl or PBS to final concentration of 0.3 nM. The resultant dsDNA solution was used to fill the nanopipette and the nanopipette dipped into either a 0.1M KCl +50% (w/v) PEG 35K (A) or 0.1M LiCl +50% (w/v) PEG 35K (B). A voltage of -500 mV was applied in all cases to perform dsDNA translocation. N indicates the number of translocation events detected.

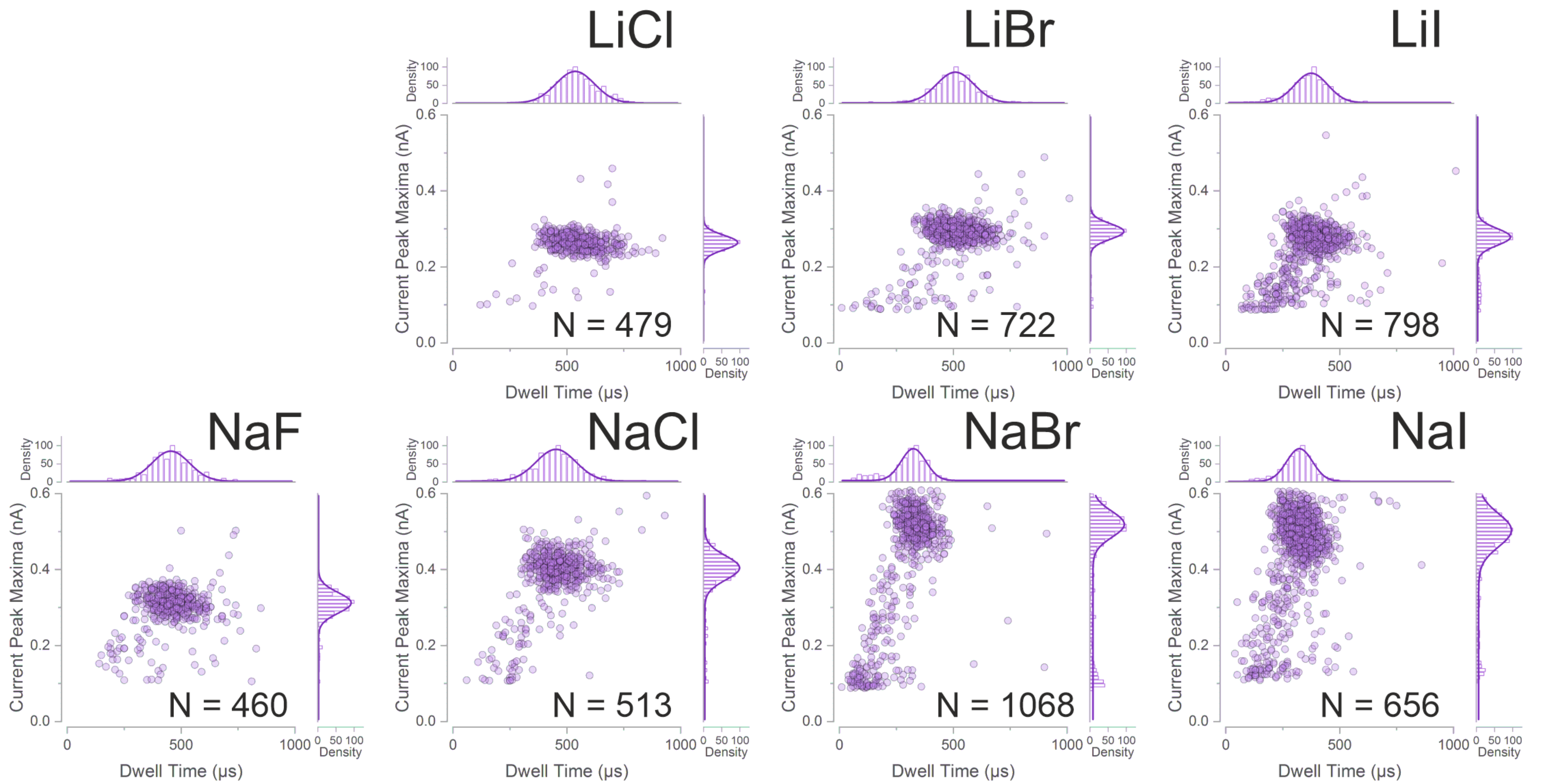

Supporting Figure 9. Population scatter plots for events recorded when dsDNA was translocated into either a lithium halide or sodium halide electrolyte bath containing PEG 35K. All nanopipettes were filled with 0.3 nM of the 4.8 kbp dsDNA diluted in 0.1M KCl. The nanopipettes were dipped into baths containing 0.1 M lithium halide +50% (w/v) PEG 35K or 0.1 M sodium halide +50% (w/v) PEG 35K electrolyte bath. The metal halide used is indicated on the upper right corner of each graph. Translocation of the dsDNA was performed by applying a voltage of -500 mV to cause the dsDNA to migrate from the nanopipette to the bath. The graphs are organised such that the halide atomic number/mass increase from left to right (from fluoride to iodide). N is the number of data plotted.

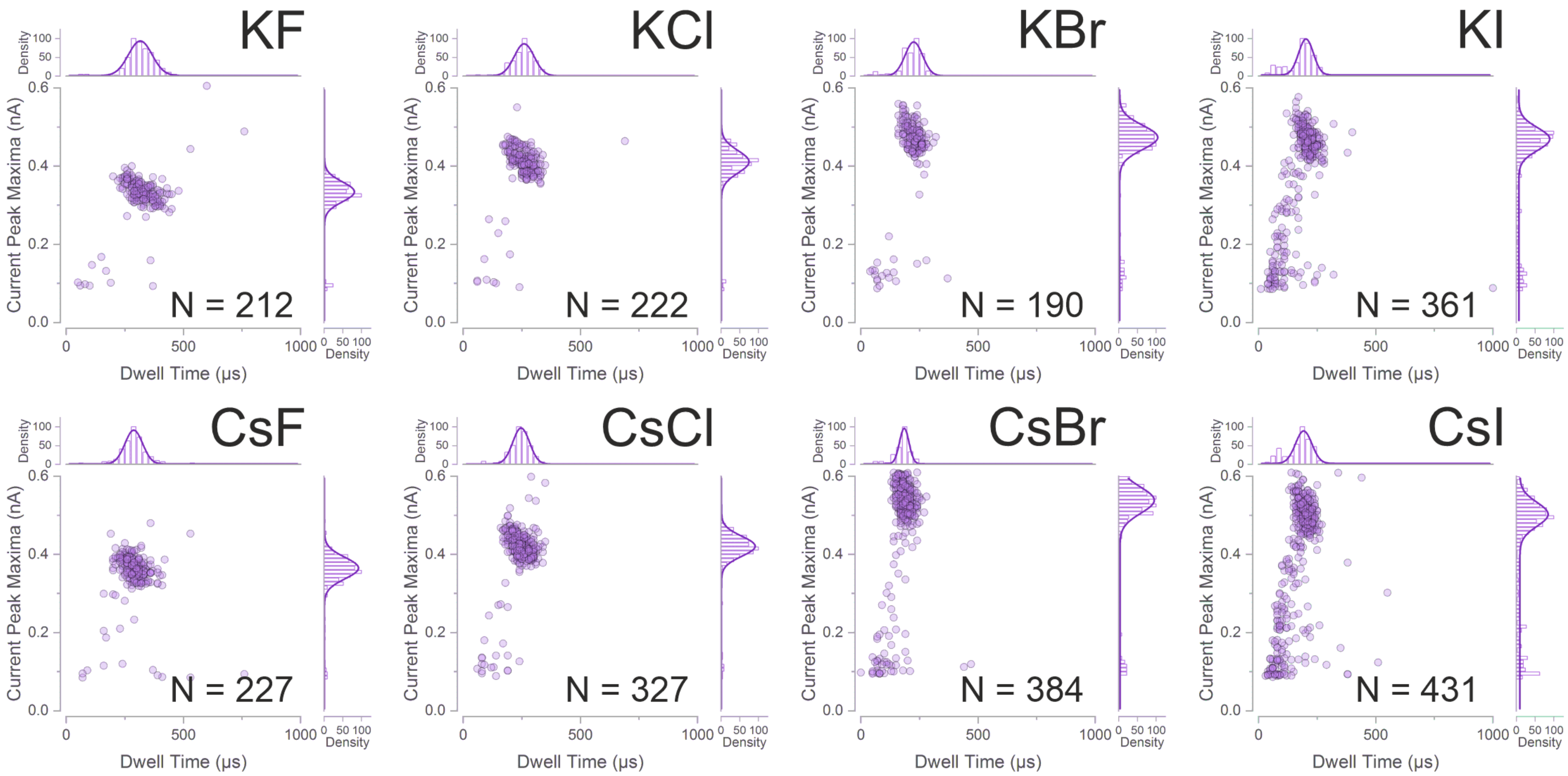

Supporting Figure 10. Population scatter plots for events recorded when dsDNA was translocated into either a potassium halide or caesium halide electrolyte bath containing PEG 35K. All nanopipettes were filled with 0.3 nM of the 4.8 kbp dsDNA diluted in 0.1M KCl. The nanopipettes were dipped into baths containing 0.1 M potassium halide +50% (w/v) PEG 35K or 0.1 M caesium halide +50% (w/v) PEG 35K electrolyte bath. The metal halide used is indicated on the upper right corner of each graph. Translocation of the dsDNA was performed by applying a voltage of -500 mV to cause the dsDNA to migrate from the nanopipette to the bath. The graphs are organised such that the halide atomic number/mass increase from left to right (from fluoride to iodide). N is the number of data plotted.

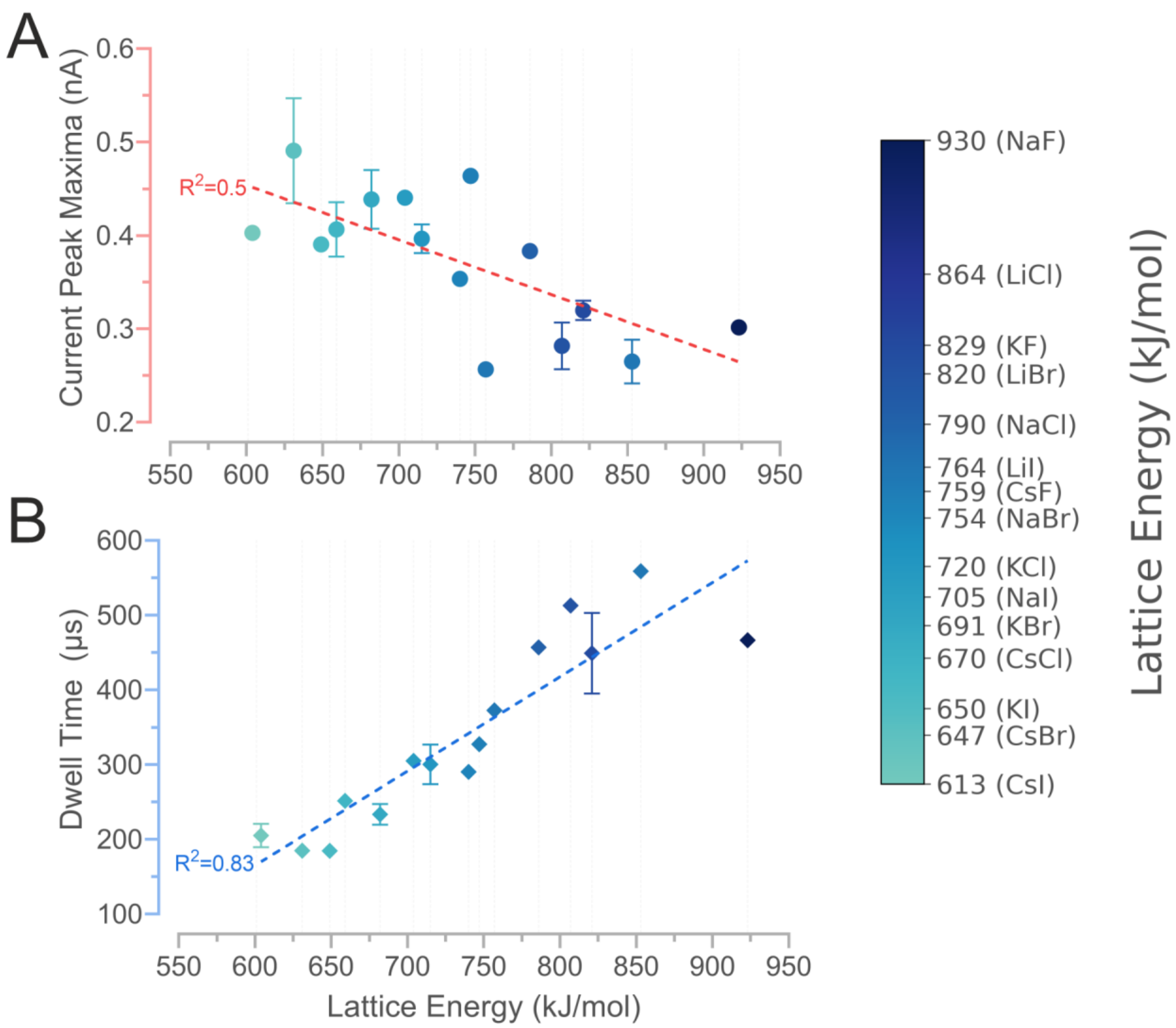

Supporting Figure 11. The linear regression plots of the current and the dwell time against the lattice energy of the alkali metal halides. The average dwell time (A) and current peak maxima (B) for translocation events for dsDNA translocated into 0.1M alkali metal halide +50% (w/v) PEG 35K electrolyte baths plotted against the lattice energy for the salts. Linear regressions were performed on both data set, the R^2 indicates the coefficient of determination.

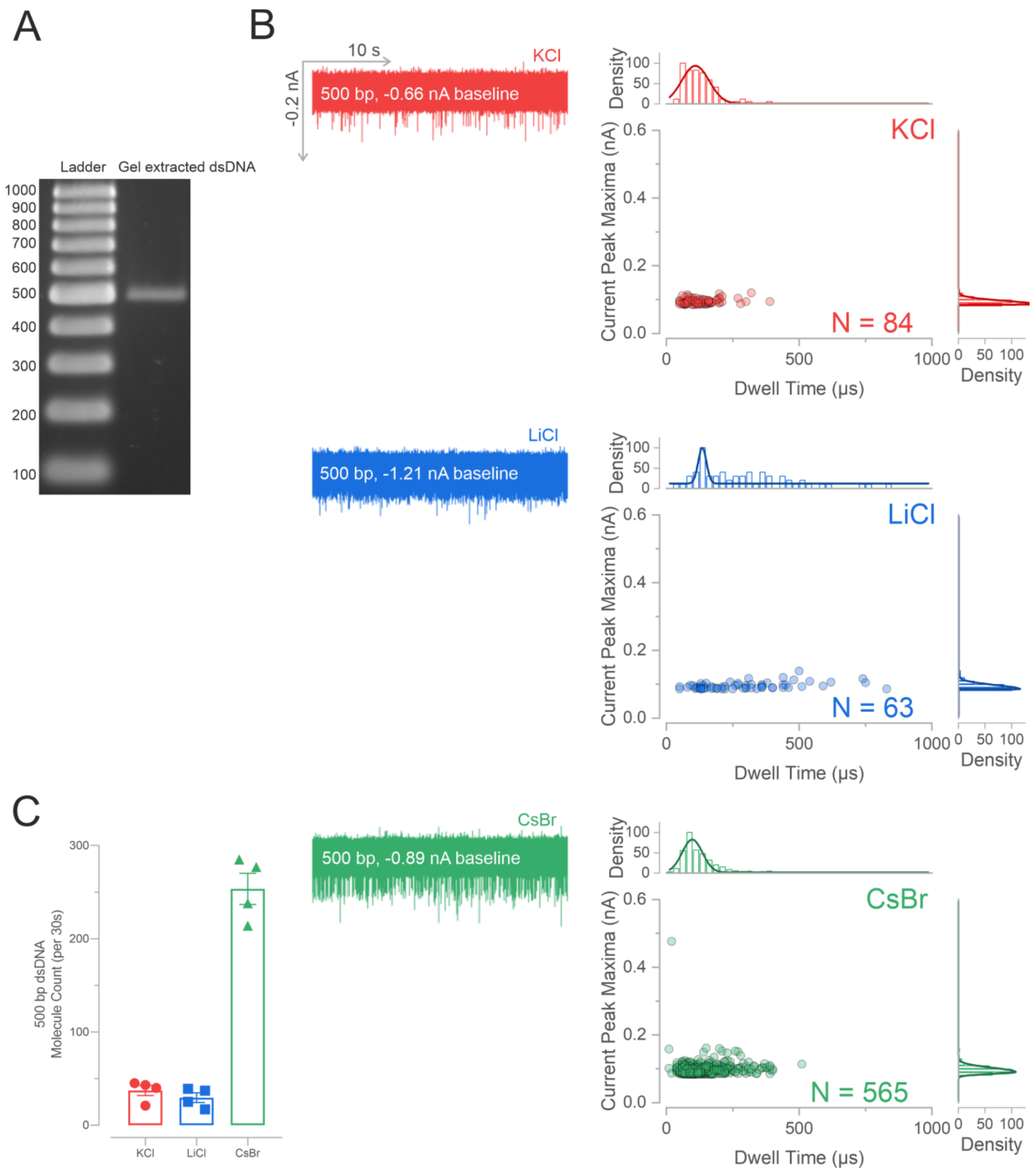

Supporting Figure 12. Nanopore analysis of the 500 bp dsDNA translocated into a PEG electrolyte bath containing different halide salts. (A) 1 µg of the gel extracted 500 bp dsDNA was analysed by gel electrophoresis with a 0.8% SYBR safe incorporated agarose gel. A DNA ladder (GeneRuler 100 bp (SM0241, Thermo Fisher)) was run alongside. The extracted sample migrated at ~ 500 bp relative to the DNA ladder. The 500 bp dsDNA fragment was isolated and purified from the same DNA ladder. (B) The 500 bp dsDNA was diluted in 0.1 M KCl and immersed into either the 0.1 M KCl, 0.1 M LiCl or 0.1 M CsBr PEG solution under a -500mV applied voltage. (C) CsBr had the highest current magnitude signal-to-noise ratio and consequently the number of recorded events were the higher than observed for the other salts. Four 30 seconds traces were used for analysis. Error bars are S.E.M. Each symbol represents the number of events recorded per 30 s. N is the number of events detected. The value superposed to the trace represents the average values of the ion current baseline.

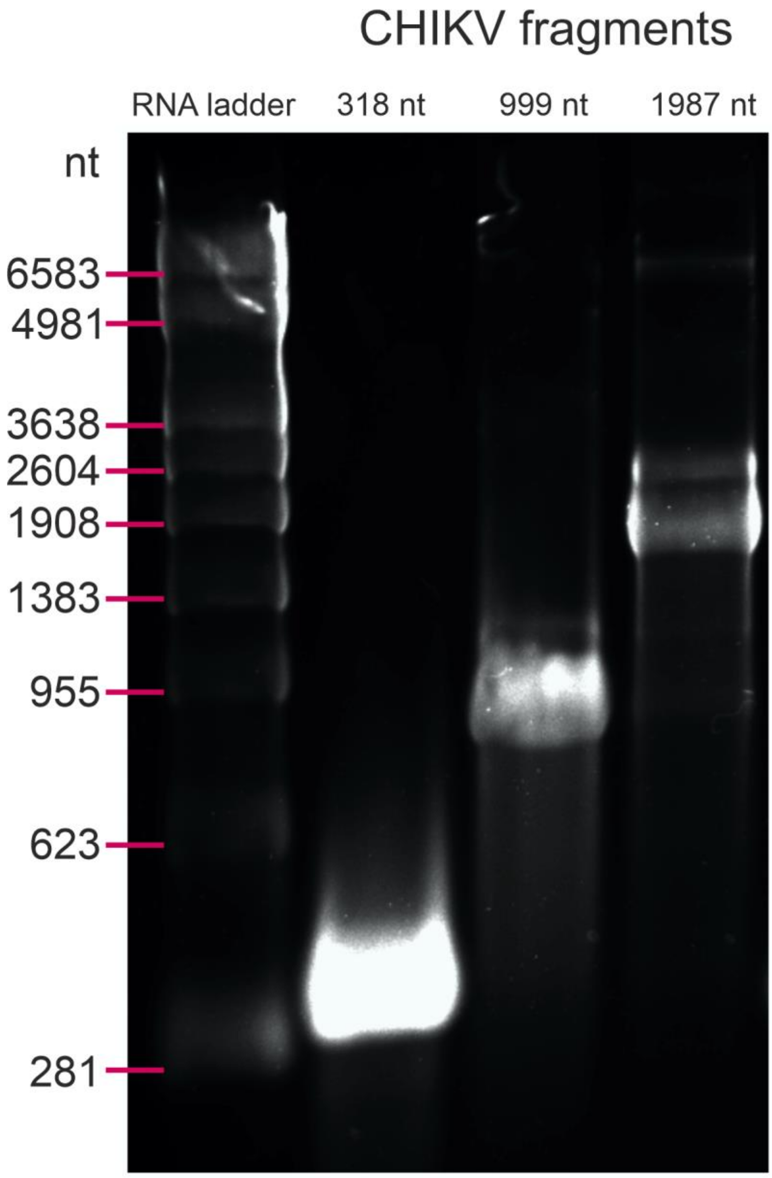

Supporting Figure 13. Analysis of *in vitro* transcribed CHIKV RNA fragments by 2% denaturing formaldehyde MOPS agarose gel electrophoresis. CHIKV RNA fragments were resolved by electrophoresis on a 2% denaturing formaldehyde MOPS agarose gel, the RNA ladder used is the RNA markers (G3191, Promega). Two bands were observed in the 1987 nt denatured RNA samples, this could be due to the incomplete denaturation of the RNA and the formation of secondary RNA structures.

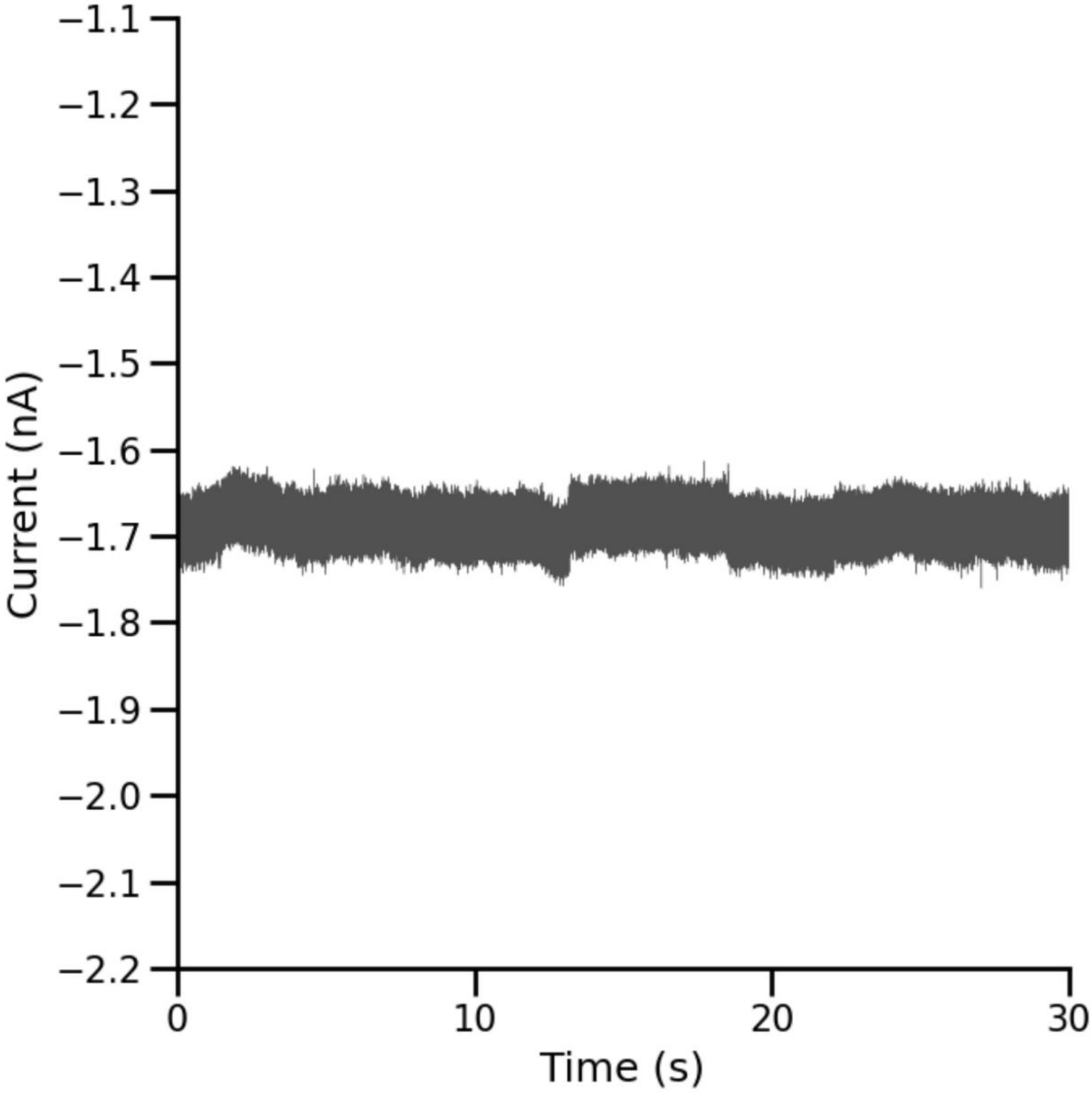

Supporting Figure 14. The translocation of 1987 nt RNA fragments into 0.1 M KCl bath, in the absence of PEG 35K. A voltage of -500 mV was used to cause the RNA to migrate from the nanopipette to the 0.1 M KCl bath. Despite some peaks were observed, no translocation peaks were called with our custom written nanopore events analysis MATLAB script, due to the stringent threshold detection level.

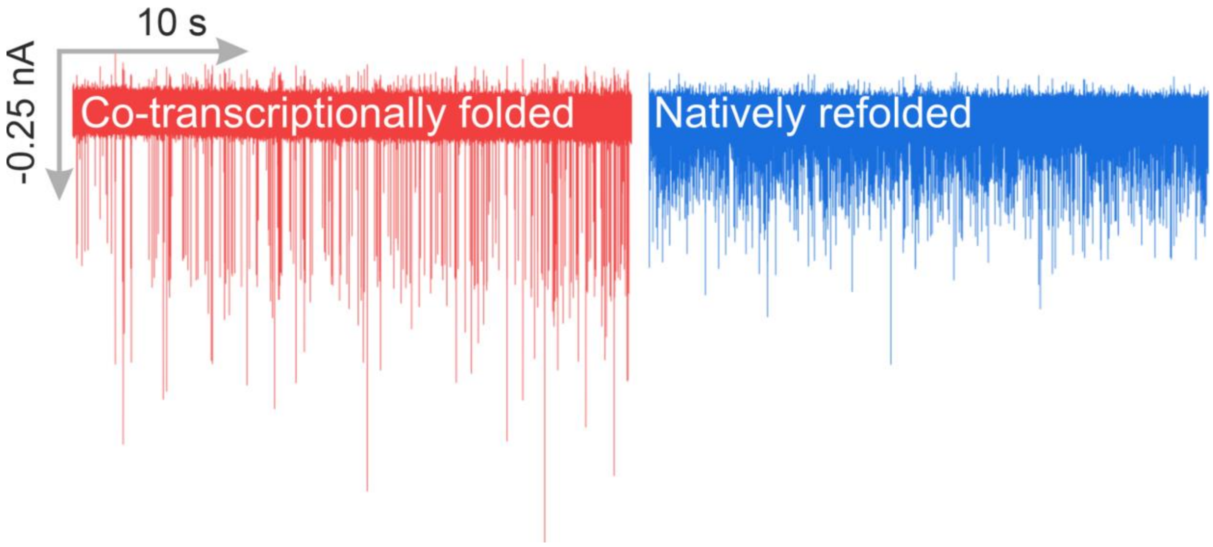

Supporting Figure 15. The translocation trace of the co-transcriptionally folded and the natively refolded 1987 CHIKV RNA. -500 mV was used to cause the RNA to translocate from nanopipette to the PEG 35K electrolyte bath.

Supporting Tables

Supporting Table 1 contains the experimental lattice energy value in kJ/mol we used in Figure 3 and Supporting Figure 11. The values can be found in the CRC handbook of chemistry and physics [1].

Supporting Table 1. The lattice energy in kJ/mol of the alkali metal halides.

Alkali metal halide	Mean dwell time (μs) ± S.E.M.	Mean Current Peak Maxima (nA) ± S.E.M.	Number of events	Lattice energy (kJ/mol)
Sodium Fluoride (NaF)	300±26	0.39±0.02	460	930
Potassium Fluoride (KF)	558±7	0.27±0.02	212	829
Caesium Fluoride (CsF)	456±7	0.38±0.003	227	759
Lithium Chloride (LiCl)	251±8	0.41±0.03	479	864
Sodium Chloride (NaCl)	467±7	0.3±0.002	513	790
Potassium Chloride (KCl)	449±54	0.32±0.01	222	720
Caesium Chloride (CsCl)	290±4	0.36±0.005	327	670
Lithium Bromide (LiBr)	512±5	0.28±0.03	722	820
Sodium Bromide (NaBr)	327±5	0.46±0.005	1068	754
Potassium Bromide (KBr)	233±13	0.44±0.03	180	691
Caesium Bromide (CsBr)	184±6	0.49±0.06	384	647
Lithium Iodide (LiI)	372±4	0.26±0.003	803	764
Sodium Iodide (NaI)	305±5	0.44±0.006	657	705
Potassium Iodide (KI)	184±4	0.39±0.007	377	650
Caesium Iodide (CsI)	205±16	0.4±0.008	431	613

Methods

Electrolyte bath preparation

The table below lists the chemicals used in the generation of the electrolyte baths.

Chemical	Catalogue	Supplier
Sodium Fluoride (NaF)	11439933	Fisher Scientific
Potassium Fluoride (KF)	10318390	Fisher Scientific
Caesium Fluoride (CsF)	012885	Alfa Aesar
Lithium Chloride (LiCl)	CHE2360	Scientific Laboratory Supplies
Sodium Chloride (NaCl)	S/3160/53	Fisher Scientific
Potassium Chloride (KCl)	P/4240/60	Fisher Scientific
Caesium Chloride (CsCl)	C3032	Sigma Aldrich
Lithium Bromide (LiBr)	013408	Alfa Aesar
Sodium Bromide (NaBr)	793574	Sigma Aldrich
Potassium Bromide (KBr)	A16339	Alfa Aesar
Caesium Bromide (CsBr)	012928	Alfa Aesar
Lithium Iodide (LiI)	044159	Alfa Aesar
Sodium Iodide (NaI)	10032620	Fisher Scientific
Potassium Iodide (KI)	PHR1360	Sigma Aldrich
Caesium Iodide (CsI)	11954211	Fisher Scientific
Dulbecco's Phosphate buffered saline (PBS)	D8537	Sigma Aldrich
Poly(ethylene) Glycol 4,000 (PEG 4K)	A16151	Alfa Aesar
Poly(ethylene) Glycol 8,000 (PEG 8K)	043443	Alfa Aesar
Poly(ethylene) Glycol 12,000 (PEG 12K)	042635	Alfa Aesar
Poly(ethylene) Glycol 35,000 (PEG 35K)	94646	Sigma Aldrich

To generate the electrolyte bath, a similar process was used to that described previously [2]. The alkali metal halide salt was dissolved to 1M with 18.2MΩ ddH$_2$O, the solution was then filtered through a 0.22 µm syringe membrane filter (E4780-1223; Starlab UK). The 1M salt solution was then diluted to 0.1M with 18.2MΩ ddH$_2$O followed by 0.22 µm filtration to generate the electrolyte bath. To generate 10 ml of the 50% (w/v) poly(ethylene) glycol (PEG), 1 ml of the 0.22 µm filtered 1M salt solution, 4 ml of 0.22 µm filtered 18.2 MΩ ddH$_2$O and 5g of PEG were mixed inside a tube. The tube was then left inside a 70°C incubator for 2 hours followed by

incubating at 37°C overnight. The tubes were then left on bench for 4 hours to reach the room temperature prior to use. All electrolytes were stored at room temperature.

dsDNA generation

To prepare the 4.8 kbp dsDNA used in this study, the plasmid pmCherry-NLS (Addgene plasmid # 39319; http://n2t.net/addgene:39319 ; RRID: Addgene_39319) was linearized by restriction digestion using the enzyme BamHI-HF (R3136; New England Biolabs) with the CutSmart Buffer (B7204S; New England Biolabs) according to the manufacturer's instruction. The linearized DNA was further purified by the Genomic DNA clean and Concentrator kit (D4010; Zymo Research) according to the manufacturer's instructions and then eluted in the Monarch® DNA Elution Buffer (T1016L; new England BioLabs Inc.). 1 µg of the linearized plasmid was analysed by gel electrophoresis with a 1% agarose gel with 1× SYBR safe dye (S33102; Thermo Fisher) incorporated into the gel. 1 µg of TrackIt™ 1 Kb Plus DNA Ladder (10488085; Thermo Fisher) was run alongside the dsDNA samples.

The 500 bp dsDNA was extracted from the GeneRuler 1 kb Plus DNA Ladder (SM1331; Thermo Fisher), which comprises fifteen chromatography-purified linear dsDNA fragments according to the manufacturer. The DNA ladder was separated via a 0.8% agarose gel, the 500 bp band was cut out of the gel and extracted using the Monarch® DNA Gel Extraction Kit as per the manufacturer's instructions (T1020; New England BioLabs Inc.).

All dsDNA were further purified using the Genomic clean and Concentrator kit (D4010; Zymo Research). The dsDNA was eluted into the Monarch® DNA Elution Buffer (T1016L; new England BioLabs Inc.) and stored at -20°C. The concentration of the resultant DNA stock solution was measured by UV absorbance at 260 nm with a NanoDrop™ 2000 (Thermo Fisher). All the dsDNA was then diluted from stock solutions to 0.3 nM with either 0.1M KCl (P/4240/60; Fisher Scientific), 0.1M LiCl (CHE2360; Scientific Laboratory Supplies) or PBS (D8537; Sigma Aldrich) prior to use.

RNA synthesis and quality control

The RNA used in this study was generated by *in vitro* transcription. The CHIKV infectious clone and sub-genomic replicons used in this study were derived from the LR2006_OPY1 La Reunion island isolate of the ECSA genotype (accession number DQ443544)[3]. The table below shows the primers used to generate the CHIKV 318, 999 and 1987 nt DNA templates.

RNA length	Forward	Reverse
318	TAATACGACTCACTATAGGGATGGCTGCGTGAGACACACG	CGCACTGCGCATCGGGCAGA
999	TAATACGACTCACTATAGGGATGGCTGCGTGAGACACACG	CATCAGGAATCCGTCTGCGT
1987	TAATACGACTCACTATAGGGATGGCTGCGTGAGACACACG	GCATCGCAATATGGTGTAGC

The KAPA HiFi HotStart ReadyMix PCR Kit (KR0370; Roche) was used to perform PCR with the Mastercycler Nexus GX2e (Eppendorf) with the following conditions to generate a DNA strand containing the T7 promoter sequence at the 5' region:

Step	Temperature	Time	Repeats
**Denaturation**	95°C	3 min	N/A
**Denaturation**	98°C	20 sec	35×
**Annealing**	65°C	15 sec	
**Elongation**	72°C	180 sec	
**Final Elongation**	72°C	10 min	N/A
**Hold**	4°C	Infinite	N/A

The resultant PCR products were purified with the Monarch® PCR & DNA Cleanup Kit (5 μg) (T1030; New England BioLabs Inc.). The sizes of the generated dsDNA fragments were confirmed by agarose gel electrophoresis. 1 μg DNA fragments were then used as templates for *in vitro* RNA transcription using the T7-Scribe™ Standard RNA IVT Kit (C-AS3107; Tebu-bio) the reaction mixture was generated according to manufacturer's instruction, followed by incubation at 37°C for 3 hours. The reaction mixture was treated with DNAse I for 30 minutes at 37°C. The RNA products were purified via phenol:chloroform purification with phenol:chloroform:isoamylalcohol (AM9730; ThermoFisher). The RNAs were then further washed and precipitated with 70% ethanol.

The synthesized RNA length was confirmed by denaturing formaldehyde MOPS (3-(*N*-morpholino)propanesulfonic acid) gel running in a 1×MOPS buffer (0.04 M MOPS at pH 7.0, 0.01 M sodium acetate, 0.001 M EDTA at pH 8.0). The 2% formaldehyde MOPS gel contained 2%

(w/v) agarose, 0.04 M MOPS at pH 7.0, 0.01 M sodium acetate, 0.001 M EDTA at pH 8.0 and 10% (v/v) formaldehyde (37% stock). 2 µg of the RNAs was mixed with RNA loading dye (B0363S; New England BioLabs Inc.), followed by incubation at 70°C for 10 minutes, then loaded into the solidified formaldehyde MOPS gel. The RNA Markers (G3191; Promega) RNA ladder, were run alongside the samples.

To refold the RNA into its native conformation, 13.4 µl of RNA samples were mixed with 6.6 µl of RNA folding buffer (333 mM HEPES at pH 8.0, 20 mM $MgCl_2$, 333 mM NaCl). The RNAs were refolded by 3 steps inside a thermocycler: 95°C for 2 mins followed by 4°C for 2 mins followed by 37°C for 30 mins. After refolding, the RNAs was kept on ice and analysed within 30 mins after refolding.

RNA synthesis related sequence

Below shows the sequence of the three DNA templates used to generate the RNA fragments:

CHIKV 318 nt

5′-

TAATACGACTCACTATAGGGATGGCTGCGTGAGACACACGTAGCCTACCAGTTTCTTACTGCTCTACTCTGCAAAGCAAGAGATTAATAACCCA
TCATGGATCCTGTGTACGTGGACATAGACGCTGACAGCGCCTTTTTGAAGGCCCTGCAACGTGCGTACCCCATGTTTGAGGTGGAACCAAGGCA
GGTCACACCGAATGACCATGCTAATGCTAGAGCGTTCTCGCATCTAGCTATAAAACTAATAGAGCAGGAAATTGACCCCGACTCAACCATCCTG
GATATCGGCAGTGCGCCAGCAAGGAGGATGATGTCGGACAGGAAGTACCACTG

-3′

T7 Promoter

CHIKV 999 nt

5′-

TAATACGACTCACTATAGGGATGGCTGCGTGAGACACACGTAGCCTACCAGTTTCTTACTGCTCTACTCTGCAAAGCAAGAGATTAATAACCCA
TCATGGATCCTGTGTACGTGGACATAGACGCTGACAGCGCCTTTTTGAAGGCCCTGCAACGTGCGTACCCCATGTTTGAGGTGGAACCAAGGCA
GGTCACACCGAATGACCATGCTAATGCTAGAGCGTTCTCGCATCTAGCTATAAAACTAATAGAGCAGGAAATTGACCCCGACTCAACCATCCTG
GATATCGGCAGTGCGCCAGCAAGGAGGATGATGTCGGACAGGAAGTACCACTGCGTCTGCCCGATGCGCAGTGCGGAAGATCCCGAGAGACTCG
CCAATTATGCGAGAAAGCTAGCATCTGCCGCAGGAAAAGTCCTGGACAGAAACATCTCTGGAAAGATCGGGGACTTACAAGCAGTAATGGCCGT
GCCAGACACGGAGACGCCAACATTCTGCTTACACACAGACGTCTCATGTAGACAGAGAGCAGACGTCGCTATATACCAAGACGTCTATGCTGTA
CACGCACCCACGTCGCTATACCACCAGGCGATTAAAGGGGTCCGAGTGGCGTACTGGGTTGGGTTCGACACAACCCCGTTCATGTACAATGCCA
TGGCGGGTGCCTACCCCTCATACTCGACAAACTGGGCAGATGAGCAGGTACTGAAGGCTAAGAACATAGGATTATGTTCAACAGACCTGACGGA
AGGTAGACGAGGCAAGTTGTCTATTATGAGAGGGAAAAAGCTAAAACCGTGCGACCGTGTGCTGTTCTCAGTAGGGTCAACGCTCTACCCGGAA
AGCCGCAAGCTACTTAAGAGCTGGCACCTGCCATCGGTGTTCCATTTAAAGGGCAAACTCAGCTTCACATGCCGCTGTGATACAGTGGTTTCGT
GTGAGGGCTACGTCGTTAAGAGAATAACGATGAGCCCAGGCCTTTATGGAAAAACCACAGGGTATGCGGTAACCCA

-3′

T7 Promoter

CHIKV 1987 nt

5′-

TAATACGACTCACTATAGGGATGGCTGCGTGAGACACACGTAGCCTACCAGTTTCTTACTGCTCTACTCTGCAAAGCAAGAGATTAATAACCCA
TCATGGATCCTGTGTACGTGGACATAGACGCTGACAGCGCCTTTTTGAAGGCCCTGCAACGTGCGTACCCCATGTTTGAGGTGGAACCAAGGCA
GGTCACACCGAATGACCATGCTAATGCTAGAGCGTTCTCGCATCTAGCTATAAAACTAATAGAGCAGGAAATTGACCCCGACTCAACCATCCTG
GATATCGGCAGTGCGCCAGCAAGGAGGATGATGTCGGACAGGAAGTACCACTGCGTCTGCCCGATGCGCAGTGCGGAAGATCCCGAGAGACTCG
CCAATTATGCGAGAAAGCTAGCATCTGCCGCAGGAAAAGTCCTGGACAGAAACATCTCTGGAAAGATCGGGGACTTACAAGCAGTAATGGCCGT
GCCAGACACGGAGACGCCAACATTCTGCTTACACACAGACGTCTCATGTAGACAGAGAGCAGACGTCGCTATATACCAAGACGTCTATGCTGTA
CACGCACCCACGTCGCTATACCACCAGGCGATTAAAGGGGTCCGAGTGGCGTACTGGGTTGGGTTCGACACAACCCCGTTCATGTACAATGCCA
TGGCGGGTGCCTACCCCTCATACTCGACAAACTGGGCAGATGAGCAGGTACTGAAGGCTAAGAACATAGGATTATGTTCAACAGACCTGACGGA
AGGTAGACGAGGCAAGTTGTCTATTATGAGAGGGAAAAAGCTAAAACCGTGCGACCGTGTGCTGTTCTCAGTAGGGTCAACGCTCTACCCGGAA
AGCCGCAAGCTACTTAAGAGCTGGCACCTGCCATCGGTGTTCCATTTAAAGGGCAAACTCAGCTTCACATGCCGCTGTGATACAGTGGTTTCGT
GTGAGGGCTACGTCGTTAAGAGAATAACGATGAGCCCAGGCCTTTATGGAAAAACCACAGGGTATGCGGTAACCCACCACGCAGACGGATTCCT
GATGTGCAAGACTACCGACACGGTTGACGGCGAAAGAATGTCATTCTCGGTGTGCACATACGTGCCGGCGACCATTTGTGATCAAATGACCGGC
ATCCTTGCTACAGAAGTCACGCCGGAGGATGCACAGAAGCTGTTGGTGGGGCTGAACCAGAGAATAGTGGTTAACGGCAGAACGCAACGGAATA

CGAACACCATGAAAAATTATCTGCTTCCCGTGGTCGCCCAAGCCTTCAGTAAGTGGGCAAAGGAGTGCCGGAAAGACATGGAAGATGAAAAACT
CCTGGGGGTCAGAGAAAGAACACTGACCTGCTGCTGTCTATGGGCATTCAAGAAGCAGAAAACACACACGGTCTACAAGAGGCCTGATACCCAG
TCAATTCAGAAGGTTCAGGCCGAGTTTGACAGCTTTGTGGTACCGAGTCTGTGGTCGTCCGGGTTGTCAATCCCTTTGAGGACTAGAATCAAAT
GGTTGTTAAGCAAGGTGCCAAAAACCGACCTGATCCCATACAGCGGAGACGCCCGAGAAGCCCGGGACGCAGAAAAAGAAGCAGAGGAAGAACG
AGAAGCAGAACTGACTCGCGAAGCCCTACCACCTCTACAGGCAGCACAGGAAGATGTTCAGGTCGAAATCGACGTGGAACAGCTTGAGGACAGA
GCGGGCGCAGGAATAATAGAGACTCCGAGAGGAGCTATCAAAGTTACTGCCCAACCAACAGACCACGTCGTGGGAGAGTACCTGGTACTCTCCC
CGCAGACCGTACTACGTAGCCAGAAGCTCAGTCTGATTCACGCTTTGGCGGAGCAAGTGAAGACGTGCACGCACAACGGACGAGCAGGGAGGTA
TGCGGTCGAAGCGTACGACGGCCGAGTCCTAGTGCCCTCAGGCTATGCAATCTCGCCTGAAGACTTCCAGAGTCTAAGCGAAAGCGCAACGATG
GTGTATAACGAAAGAGAGTTCGTAAACAGA

-3′

T7 Promoter

Below shows the sequence of the three RNA fragments generated via *in vitro* transcription with T7 RNA polymerase:

CHIKV 318 nt

5′-

GGGAUGGCUGCGUGAGACACACGUAGCCUACCAGUUUCUUACUGCUCUACUCUGCAAAGCAAGAGAUUAAUAACCCAUCAUGGAUCCUGUGUAC
GUGGACAUAGACGCUGACAGCGCCUUUUUGAAGGCCCUGCAACGUGCGUACCCCAUGUUUGAGGUGGAACCAAGGCAGGUCACACCGAAUGACC
AUGCUAAUGCUAGAGCGUUCUCGCAUCUAGCUAUAAAACUAAUAGAGCAGGAAAUUGACCCCGACUCAACCAUCCUGGAUAUCGGCAGUGCGCC
AGCAAGGAGGAUGAUGUCGGACAGGAAGUACCACUG

-3′

CHIKV 999 nt

5′-

GGGAUGGCUGCGUGAGACACACGUAGCCUACCAGUUUCUUACUGCUCUACUCUGCAAAGCAAGAGAUUAAUAACCCAUCAUGGAUCCUGUGUAC
GUGGACAUAGACGCUGACAGCGCCUUUUUGAAGGCCCUGCAACGUGCGUACCCCAUGUUUGAGGUGGAACCAAGGCAGGUCACACCGAAUGACC
AUGCUAAUGCUAGAGCGUUCUCGCAUCUAGCUAUAAAACUAAUAGAGCAGGAAAUUGACCCCGACUCAACCAUCCUGGAUAUCGGCAGUGCGCC
AGCAAGGAGGAUGAUGUCGGACAGGAAGUACCACUGCGUCUGCCCGAUGCGCAGUGCGGAAGAUCCCGAGAGACUCGCCAAUUAUGCGAGAAAG
CUAGCAUCUGCCGCAGGAAAAGUCCUGGACAGAAACAUCUCUGGAAAGAUCGGGGACUUACAAGCAGUAAUGGCCGUGCCAGACACGGAGACGC
CAACAUUCUGCUUACACACAGACGUCUCAUGUAGACAGAGAGCAGACGUCGCUAUAUACCAAGACGUCUAUGCUGUACACGCACCCACGUCGCU
AUACCACCAGGCGAUUAAAGGGGUCCGAGUGGCGUACUGGGUUGGGUUCGACACAACCCCGUUCAUGUACAAUGCCAUGGCGGGUGCCUACCCC
UCAUACUCGACAAACUGGGCAGAUGAGCAGGUACUGAAGGCUAAGAACAUAGGAUUAUGUUCAACAGACCUGACGGAAGGUAGACGAGGCAAGU
UGUCUAUUAUGAGAGGGAAAAAGCUAAAACCGUGCGACCGUGUGCUGUUCUCAGUAGGGUCAACGCUCUACCCGGAAAGCCGCAAGCUACUUAA
GAGCUGGCACCUGCCAUCGGUGUUCCAUUUAAAGGGCAAACUCAGCUUCACAUGCCGCUGUGAUACAGUGGUUUCGUGUGAGGGCUACGUCGUU
AAGAGAAUAACGAUGAGCCCAGGCCUUUAUGGAAAAACCACAGGGUAUGCGGUAACCCA

-3′

CHIKV 1987 nt

5′-

GGGAUGGCUGCGUGAGACACACGUAGCCUACCAGUUUCUUACUGCUCUACUCUGCAAAGCAAGAGAUUAAUAACCCAUCAUGGAUCCUGUGUAC
GUGGACAUAGACGCUGACAGCGCCUUUUUGAAGGCCCUGCAACGUGCGUACCCCAUGUUUGAGGUGGAACCAAGGCAGGUCACACCGAAUGACC
AUGCUAAUGCUAGAGCGUUCUCGCAUCUAGCUAUAAAACUAAUAGAGCAGGAAAUUGACCCCGACUCAACCAUCCUGGAUAUCGGCAGUGCGCC
AGCAAGGAGGAUGAUGUCGGACAGGAAGUACCACUGCGUCUGCCCGAUGCGCAGUGCGGAAGAUCCCGAGAGACUCGCCAAUUAUGCGAGAAAG
CUAGCAUCUGCCGCAGGAAAAGUCCUGGACAGAAACAUCUCUGGAAAGAUCGGGGACUUACAAGCAGUAAUGGCCGUGCCAGACACGGAGACGC
CAACAUUCUGCUUACACACAGACGUCUCAUGUAGACAGAGAGCAGACGUCGCUAUAUACCAAGACGUCUAUGCUGUACACGCACCCACGUCGCU

AUACCACCAGGCGAUUAAAGGGGUCCGAGUGGCGUACUGGGUUGGGUUCGACACAACCCCGUUCAUGUACAAUGCCAUGGCGGGUGCCUACCCC

UCAUACUCGACAAACUGGGCAGAUGAGCAGGUACUGAAGGCUAAGAACAUAGGAUUAUGUUCAACAGACCUGACGGAAGGUAGACGAGGCAAGU

UGUCUAUUAUGAGAGGGAAAAAGCUAAAACCGUGCGACCGUGUGCUGUUCUCAGUAGGGUCAACGCUCUACCCGGAAAGCCGCAAGCUACUUAA

GAGCUGGCACCUGCCAUCGGUGUUCCAUUUAAAGGGCAAACUCAGCUUCACAUGCCGCUGUGAUACAGUGGUUUCGUGUGAGGGCUACGUCGUU

AAGAGAAUAACGAUGAGCCCAGGCCUUUAUGGAAAAACCACAGGGUAUGCGGUAACCCACCACGCAGACGGAUUCCUGAUGUGCAAGACUACCG

ACACGGUUGACGGCGAAAGAAUGUCAUUCUCGGUGUGCACAUACGUGCCGGCGACCAUUUGUGAUCAAAUGACCGGCAUCCUUGCUACAGAAGU

CACGCCGGAGGAUGCACAGAAGCUGUUGGUGGGGCUGAACCAGAGAAUAGUGGUUAACGGCAGAACGCAACGGAAUACGAACACCAUGAAAAAU

UAUCUGCUUCCCGUGGUCGCCCAAGCCUUCAGUAAGUGGGCAAAGGAGUGCCGGAAAGACAUGGAAGAUGAAAAACUCCUGGGGGUCAGAGAAA

GAACACUGACCUGCUGCUGUCUAUGGGCAUUCAAGAAGCAGAAAACACACACGGUCUACAAGAGGCCUGAUACCCAGUCAAUUCAGAAGGUUCA

GGCCGAGUUUGACAGCUUUGUGGUACCGAGUCUGUGGUCGUCCGGGUUGUCAAUCCCUUUGAGGACUAGAAUCAAAUGGUUGUUAAGCAAGGUG

CCAAAAACCGACCUGAUCCCAUACAGCGGAGACGCCCGAGAAGCCCGGGACGCAGAAAAAGAAGCAGAGGAAGAACGAGAAGCAGAACUGACUC

GCGAAGCCCUACCACCUCUACAGGCAGCACAGGAAGAUGUUCAGGUCGAAAUCGACGUGGAACAGCUUGAGGACAGAGCGGGCGCAGGAAUAAU

AGAGACUCCGAGAGGAGCUAUCAAAGUUACUGCCCAACCAACAGACCACGUCGUGGGAGAGUACCUGGUACUCUCCCCGCAGACCGUACUACGU

AGCCAGAAGCUCAGUCUGAUUCACGCUUUGGCGGAGCAAGUGAAGACGUGCACGCACAACGGACGAGCAGGGAGGUAUGCGGUCGAAGCGUACG

ACGGCCGAGUCCUAGUGCCCUCAGGCUAUGCAAUCUCGCCUGAAGACUUCCAGAGUCUAAGCGAAAGCGCAACGAUGGUGUAUAACGAAAGAGA

GUUCGUAAACAGA

-3′

Scanning electron microscopy

The nanopores of the nanopipettes were imaged by scanning electron microscopy (Leo 1530 FEG-SEM; Zeiss). Nanopipettes were first sputter coated with a gold layer of a few nanometres in thicknesses. The nanopipettes were then mounted onto the sample holder and tilted to an angle of nearly 90 for imaging. The nanopipettes were imaged at between 2 and 3 kV at a working distance of 5 mm and below at an aperture size of 30.00 m using an InLens detector.

References

(1) Haynes, W. M. Section 12: Properties of Solids. In *CRC handbook of chemistry and physics*, 97th ed.; CRC Press: Boca Raton, 2016; Vol. 1, pp 12-22 - 12-35.

(2) Chau, C. C.; Radford, S. E.; Hewitt, E. W.; Actis, P. Macromolecular Crowding Enhances the Detection of DNA and Proteins by a Solid-State Nanopore. *Nano Letters* **2020**, *20* (7), 5553-5561. DOI: 10.1021/acs.nanolett.0c02246.

(3) Tsetsarkin, K.; Higgs, S.; McGee, C. E.; Lamballerie, X. D.; Charrel, R. N.; Vanlandingham, D. L. Infectious Clones of Chikungunya Virus (La Réunion Isolate) for Vector Competence Studies. *Vector-Borne and Zoonotic Diseases* **2006**, *6* (4), 325-337. DOI: 10.1089/vbz.2006.6.325.
